# Antioxidant Application of Clove (*Syzygium aromaticum*) Essential Oil in Meat and Meat Products: A Systematic Review

**DOI:** 10.3390/plants14131958

**Published:** 2025-06-26

**Authors:** Eduardo Valarezo, Guicela Ledesma-Monteros, Ximena Jaramillo-Fierro, Matteo Radice, Miguel Angel Meneses

**Affiliations:** 1 Departamento de Química, Universidad Técnica Particular de Loja, Loja 1101608, Ecuador; xvjaramillo@utpl.edu.ec (X.J.-F.); mameneses@utpl.edu.ec (M.A.M.); 2 Carrera de Alimentos, Universidad Técnica Particular de Loja, Loja 1101608, Ecuador; mgledesma1@utpl.edu.ec; 3 Facultad de Ciencias de la Tierra, Universidad Estatal Amazónica, Puyo 160150, Ecuador; mradice@uea.edu.ec

**Keywords:** antioxidant activity, beef, edible films, fish, lipid peroxidation, protein oxidation

## Abstract

The essential oil isolated from clove (*Syzygium aromaticum*) is used in food, medicine, cosmetics, agriculture, and aromatherapy for its antimicrobial, antioxidant, and analgesic properties. This systematic review, following the PRISMA 2020 methodology, evaluates the application of clove essential oil in meat and meat products to determine its effectiveness in preventing oxidative damage and improving product quality. A search was performed in various databases, obtaining 639 studies. After removing duplicates and applying inclusion and exclusion criteria, 43 relevant articles were selected. Studies published between 1999 and 2024 that evaluated clove essential oil in meat for human consumption were included, excluding research on extracts other than essential oil or supplements for animal feed. The studies suggest that clove essential oil improves parameters such as oxidative stability, colour preservation, and the reduction in reactive compounds such as thiobarbituric acid-reactive substances, thereby increasing the shelf life and safety of meat and meat products. Oxidation is reduced through free radical inhibition and lipid protection. The main variability detected includes the type of meat, application method and storage conditions. The concentrations used ranged from 2.65 mL/kg to 5%. Although variability in methodologies and concentrations used is a limitation for meta-analysis, the findings support the potential of clove essential oil as a natural alternative for preserving meat products, responding to consumer demand for safer foods free of synthetic preservatives.

## 1. Introduction

The meat industry, as an integral part of the global food chain, constantly faces the challenge of ensuring the quality and safety of its meat products [1]. In a context in which the demand for healthier and more sustainable foods is constantly increasing, plant-based solutions have become a promising alternative to reducing dependence on chemical additives in the production and preservation of meat and meat products. Thus, among the natural ingredients that have attracted the attention of researchers and professionals in the meat industry are compounds of plant origin [2,3]. Essential oils (EOs) are a mixture of volatile compounds extracted from various plant parts [4]. These compounds, characterised by their aromatic properties, have become the subject of interdisciplinary research ranging from organic chemistry and botany to medicine and psychology [5]. The five main plant families known for producing EOs are Apiaceae, Asteraceae, Lamiaceae, Myrtaceae, and Rutaceae. The Myrtaceae family features species like eucalyptus and clove, widely used for their biological properties [6].

*Syzygium aromaticum* (L.) Merr. & L.M. Perry, commonly known as clove, is an interesting plant species with potential as a food preservative, even being evaluated for applications in active food packaging [7,8]. The phytochemical analysis of various types of clove extract has revealed the presence of different chemical groups, such as phenolic compounds, sesquiterpenes, and monoterpenes. Clove essential oil (CEO), derived from the dried buds of the clove, stands out for its distinctive, warm, and spicy aroma. CEO for industrial purposes is mainly extracted by conventional techniques such as steam distillation and hydro-distillation. However, there are studies that cite experiments using less common techniques, such as cold-pressing, organic solvent extraction, supercritical fluid extraction, ultrasound-assisted extraction (UAE), and microwave-assisted extraction (MAE) [9,10]. This oil is characterised by its well-documented richness in bioactive compounds, many of which have antimicrobial and antioxidant properties [11]. Indeed, the literature shows several studies on the effects of CEO on the microbiological, sensory, and nutritional quality of meat and meat products [12]. Eugenol, eugenyl acetate, and caryophyllene have been reported as the main compounds in CEO [11,13], and these molecules strongly support its wide range of biological activities reported in the literature [4], e.g., antioxidant activity [14]. The antioxidant activity of CEO and its main compounds has been widely documented by several authors, who highlighted the oil’s strong scavenging activity against free radicals, such as superoxide and hydrogen peroxide. These results support the application of CEO as an additive for food preservation and, in general, as a natural product useful for human health [15]. The antioxidant action mechanism of eugenol, which is generally the main compound of CEO, can be traced back to the properties of phenolic compounds. The main effects of antioxidant action in foods consist of deactivating free radicals by the transfer of single electrons or hydrogen atoms. In addition, the chelation of metals involved in oxidative processes reduces oxidative damage [16,17].

Meat is the muscle tissue of slaughtered animals, composed of water, proteins, lipids, minerals, and a small proportion of carbohydrates. Meat and meat products are susceptible to quality deterioration due to their rich nutritional composition [18]. This deterioration is due to chemical and microbial changes. The most common form of chemical deterioration is lipid oxidation in meat. Oxidative processes, particularly lipid and protein oxidation, are among the primary causes of quality deterioration in meats and meat products, leading to undesirable changes in flavour, colour, texture, nutritional value, and shelf life [19]. EOs, rich in bioactive compounds such as phenolics, terpenes, and aldehydes, exert their antioxidant effects mainly through free radical scavenging mechanisms. These molecules donate hydrogen atoms or electrons to neutralise free radicals, thus interrupting oxidative chain reactions. Moreover, some essential oil (EO) components can chelate pro-oxidant metal ions, further inhibiting oxidative damage in meat matrices [20]. Incorporating EOs into meat products offers a promising natural strategy to enhance product stability and safety while meeting consumer preferences for clean-label foods.

Several authors agree that the composition of CEO is responsible for its antioxidant activity since these molecules are those that influence the reduction and elimination of free radicals present in the matrix. Ansarian et al. [21] and Ghasemi et al. [22] agree that the phenolic content present in a solution allows researchers to indicate its antioxidant potential due to the close relationship that exists between both parts. Binsi et al. [23] established that the ability to neutralise free radicals is influenced by phenolic compounds since flavonoids provide hydroxyl groups that react with radicals. The antioxidant activity of polyphenols can be explained by considering the presence of the phenolic H atom. The latter can interact with peroxyl radicals, stabilising them by resonance and generating unreactive adducts; this mechanism interrupts the propagation of the oxidative chain [24]. The composition of an EO is around 300 compounds (majorities, minorities, and traces). However, most studies emphasise the antioxidant potential of eugenol. According to the literature, this molecule is a ferric ion reducing agent with electron donor properties that allow free radicals to be neutralised and stable compounds to be formed [25]. Binsi et al. [23] point out that a eugenol molecule could reduce two or more 1,1-diphenyl-2-picrylhydrazil (DPPH) radicals, which generates eugenol dimers with two phenolic hydroxyl groups from eugenyl intermediate radicals. CEO can prevent or slow down lipid and protein oxidation reactions in meat and the formation of unwanted volatile compounds, helping preserve the nutritional and sensory quality of meat and meat products [26].

This systematic review aims to compile and analyze the available scientific evidence on the antioxidant activity of CEO in meat and meat products, with the aim of providing a comprehensive view of its effectiveness and possible applications in a meat production context for healthier and safer foods, which is in line with the demands of current consumers [27]. There are several studies on the antioxidant activity of essential oils [28,29]; however, the novelty of this review lies in its systematic and specific focus on the antioxidant application of CEO in meat and meat products. To the best of our knowledge, there is no prior systematic review addressing this topic. The available evidence is scattered and presents variable results, so this review seeks to consolidate existing information, identify research gaps and provide a solid scientific basis for future applications of CEO in the food industry.

## 2. Research Strategy

This systematic review study aimed to evaluate the application of clove (*Syzygium aromaticum*) EO in meat and meat products, following the guidelines of the PRISMA 2020 methodology [30]. The review was carried out following a series of methodical steps inspired by the Cochrane systematic review framework, with necessary modifications to address the specific focus of this study.

### 2.1. Information Sources

The literature search was conducted using several academic databases: ScienceDirect, SCOPUS, Web of Science (WOS), and PubMed. These databases were selected due to their comprehensive collections of scientific articles and their accessibility.

### 2.2. Search Strategy

To capture all relevant studies on the application of CEO in meat and meat products, a detailed search strategy was developed. The search terms used were “clove” OR “*Syzygium aromaticum*” AND “essential oil” AND “antioxidant” combined with several meat-related terms, such as “meat”, “fish”, “chicken”, and “meat products”. Boolean operators (AND, OR, NOT), phrase search, truncation, wildcard (“*”), and field code functions were used to refine the search. The search terms may appear in the title, abstract, or keywords. The search was carried out in the databases PubMed, SCOPUS, ScienceDirect, or Web of Science. Table 1 shows the additional information, such as the literature type, language, and chronology, entered according to the researchers’ criteria. This strategy allowed us to identify relevant studies.

### 2.3. Eligibility Criteria

The inclusion criteria for this review were clearly defined to ensure the relevance and quality of the selected studies. Studies that focused on the application of CEO in meat and meat products intended for human consumption were included. Research involving the use of CEO alone or in combination with other EOs was also considered. Studies that were not research articles, such as books, conference reports, editorials, etc., were excluded. Exclusion criteria included studies focusing solely on extracts other than EOs, research on animal feed supplements, and theses and dissertations unless enough articles were available. These criteria ensure that only the most relevant and high-quality studies are included in the review.

### 2.4. Study Selection

The study selection process was carried out in several stages to ensure rigor and precision. Initially, a selection was made based on the titles and abstracts of the articles identified in the search. Studies that met the inclusion criteria were selected for full-text review. To avoid the inclusion of duplicates, reference management tools, specifically the Mendeley reference manager, were used to identify and eliminate duplicate records. Study eligibility was assessed independently by two reviewers, who reviewed the full articles. Any discrepancies between reviewers were resolved through discussion and consensus, thus ensuring the inclusion of the most relevant studies.

**Figure 1:**
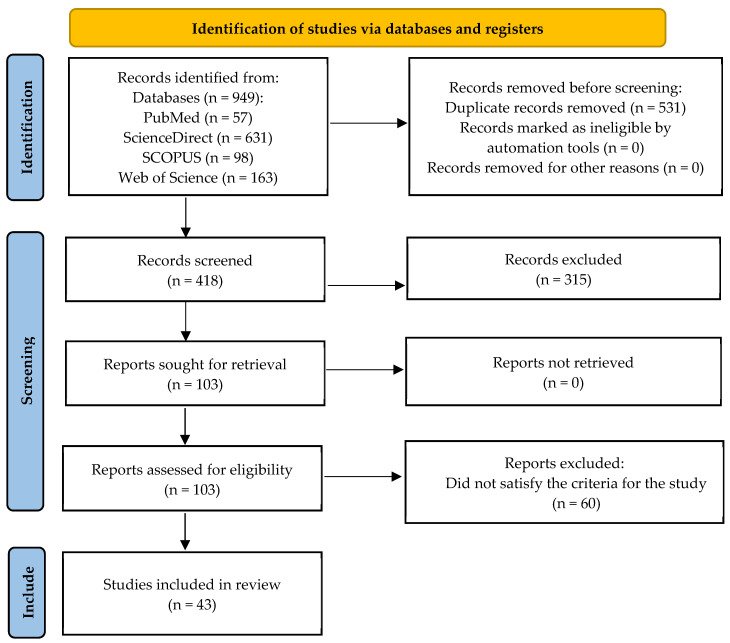
The PRISMA flowchart showing how the reviewed articles were chosen.

Searches of the PubMed, ScienceDirect, Scopus and Web of Science registers yielded 57, 631, 98, and 163 results, respectively. Following the removal of duplicates, 418 studies were screened by title and abstract, which yielded 106 studies for retrieval for full-text analysis. Among the excluded reports were those that only report the antioxidant activity of the CEO (e.g., [31]); these were excluded since the EO was not tested in meat or meat products. Reports that publish the use of EOs as food supplements for slaughtered animals (e.g., [32]) were also excluded. All these reports were retrieved, and 43 were included in the final review following full-text assessment. A PRISMA flowchart [33] depicting this process is displayed in Figure 1. The 43 journal articles included in this review are available in Supplementary Information Table S1 [21,22,23,25,34,35,36,37,38,39,40,41,42,43,44,45,46,47,48,49,50,51,52,53,54,55,56,57,58,59,60,61,62,63,64,65,66,67,68,69,70,71,72].

### 2.5. Data Extraction

Data extraction was carried out using a standardised form designed to systematically collect relevant information from each study. A total of 43 eligible articles were selected, removing duplicates and excluding 378 articles that did not meet the inclusion and exclusion criteria. Extracted data included study characteristics such as author and year of publication. Specific information was also collected on the type of meat used and the applied concentration of the EO. This systematic approach to data extraction ensures that relevant information is collected consistently and can be adequately compared across studies.

### 2.6. Quality Assessment

Two reviewers conducted the quality assessment of all selected articles, and any differences were resolved through discussion. The quality analysis was based on experimental design, methodology, application mechanisms, and results obtained.

### 2.7. Synthesis Methods

Initially, the number of products and presentations of the meat and meat product matrices were analysed. Due to the nature of the results, statistical analysis was not possible; therefore, a narrative synthesis was planned. The studies were divided into three groups based on the application of the EO: direct addition or in the form of edible films and coatings and encapsulation. Within these subgroups, the information was summarised in tables, which provide information regarding the matrix (product and presentation), main EO compounds, activity analysis method, concentration evaluated, storage conditions, results, and sensory evaluation.

The results are presented according to the method of incorporating CEO into the food matrix, that is, by direct addition, edible films or coatings and encapsulation. In each incorporation method section, the results of the different biological activities tested are detailed.

### 2.8. Limitation

Our review used the PRISMA guidelines to identify as many relevant studies as possible. The search was limited to four databases recognised for their quality and contribution to research to ensure the rigour and quality of the articles included in our evaluation. The variability in methodologies and concentrations used presented a limitation; this added to the fact that the results obtained did not allow the realisation of a meta-analysis.

## 3. Use of CEO in Meat and Meat Products

**Figure 2:**
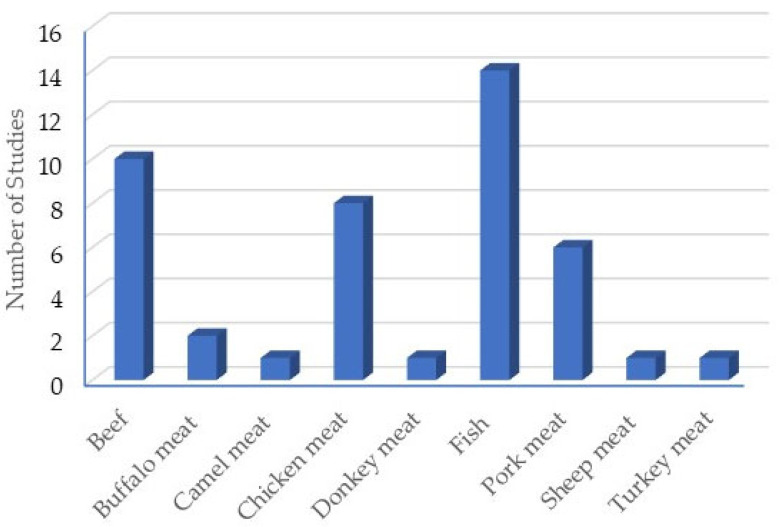
Clove essential oil application matrices.

CEO has been incorporated into nine different meat matrices. Figure 2 shows the matrices used to test clove EO and the number of studies carried out on each matrix. In total, 44 studies are shown; this is because in some articles, more than one matrix was used. The most common meat matrix for CEO testing was fish (14 studies).

**Figure 3:**
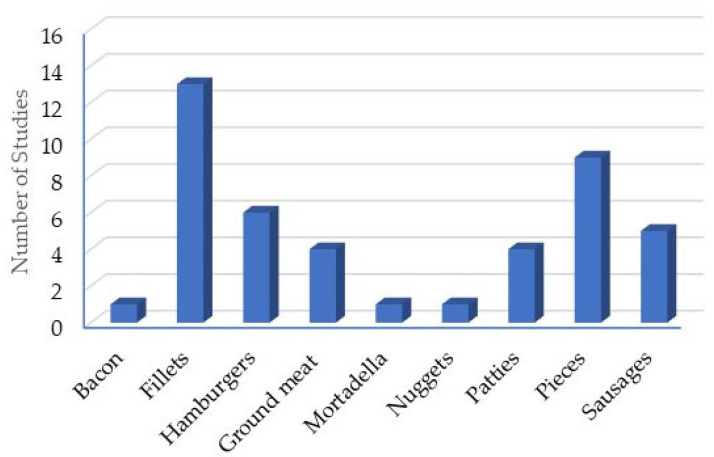
Presentation of the matrices for the application of clove essential oil.

An analysis of the presentation of the different products shows that 13 of the 40 studies were conducted on fillets (Figure 3). Among the meat products, hamburgers are featured in the highest number of articles. Hamburgers, mortadella, nuggets, patties, and sausages are considered meat products since their composition contains other ingredients besides meat.

**Figure 4:**
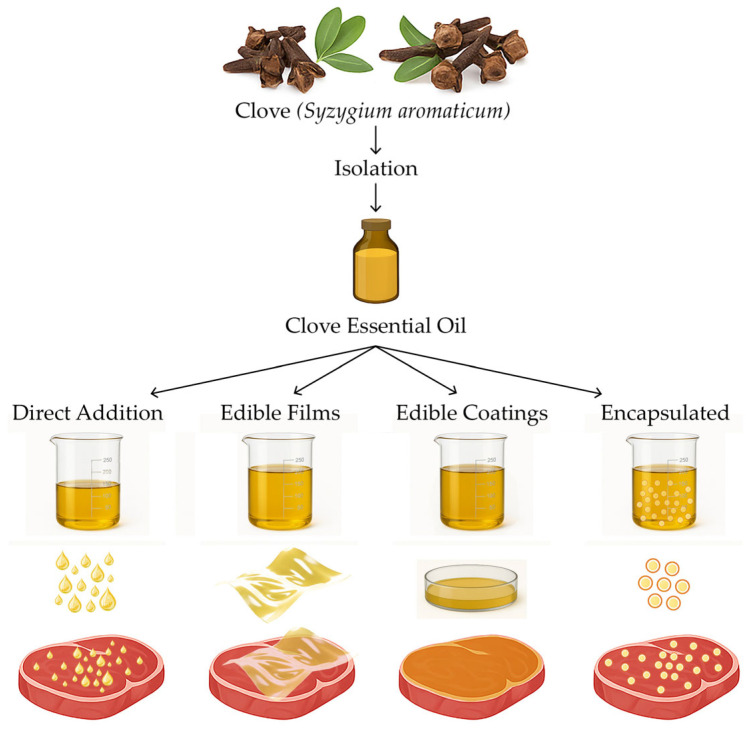
Application routes of CEO in meat and meat products.

When analysing the incorporation of CEO into meat and meat products, it was determined that there are four routes to applying it. Figure 4 shows a diagram of the incorporation of CEO into meat and meat products. In summary, 14 studies used the direct addition of CEO, 26 studies used edible films and coatings containing CEO, and 4 studies used encapsulated clove essential oil.

## 4. Direct Addition of CEO into Meat and Meat Products

Antioxidant activity in meat and meat products is crucial to preventing oxidative deterioration, which negatively affects colour, flavour, texture, and nutritional value. Reactive oxygen species (ROS) can initiate lipid and protein oxidation, leading to quality losses during storage [19]. To counteract this process, effective antioxidants are incorporated into food products. This is a simple technique that reduces lipid oxidation. Antioxidants–whether synthetic, such as butylated hydroxyanisole (BHA), butylated hydroxytoluene (BHT) and propyl gallate (PG), or natural—scavenge reactive forms of oxygen involved in oxidation [59]. Table 2 compiles the studies where CEO has been used directly as an antioxidant in meat and meat products.

Methods for determining antioxidant activity are based on verifying how an oxidising agent induces oxidative damage to an oxidisable substrate, damage that is inhibited or reduced in the presence of an antioxidant substance. This inhibition is proportional to the antioxidant activity of the compound or sample. On the other hand, there are tests based on the quantification of the products formed after the oxidative process. Zengin and Bansal [73] mentioned that the interpretation of antioxidant activity requires a combination of different data.

Authors such as Ansarian et al. [21] and Ghasemi et al. [22] reported that the total phenolic content present in a solution helps indicate its antioxidant potential due to the close relationship that exists between both parts. Sharma et al. [59] established that a higher total phenolic content is directly related to greater oxidative stability owing to the presence of bioactive compounds, which interfere with the initiation and sequential reactions that can trigger oxidative rancidity of the product. Studies have revealed promising results with respect to CEO since in all of them, a higher phenolic content, in particular eugenol, is evident compared to its counterparts like thyme, cassia, marjoram, rosemary, and sage [22,59,73].

Regarding the measurement of antioxidant activity using DPPH and ABTS methods, the samples (Table 2) treated with CEO showed more free radical scavenging activity compared to the control and other treatments with EOs. Sharma et al. [59] applied CEO to the surface of thawed chicken pieces, then cut the meat into small pieces and mixed it with the rest of the ingredients to make sausages. They found that during the first five days of treating chicken sausages with 0.25% of CEO, there was an increase in antioxidant activity due to reduced formation of free radicals. However, after this time, there was a change in pH and an increase in lipid oxidation caused by endogenous and external factors affecting the samples, so the oxidative inhibition was reduced. Meanwhile, Ghasemi et al. [22] and Sharma et al. [59] verified that DPPH values increased in chicken meat until day 5 and then decreased due to an increase in lipid oxidation.

When seeking to combine EOs and extracts with the aim of increasing antioxidant activity, it is necessary to evaluate the previous behaviour, since a synergistic effect is not always obtained but can be antagonistic or additive. For this reason, it is advisable to evaluate the type of interactions using the fractional antioxidant capacity (FAC) index, which helps simulate the experimental proportion of free radical elimination. Khodaei et al. [45] analysed the interactions between cinnamon and CEO, where an FAC value of 0.2 was evident. This revealed an antagonistic effect; as a consequence, oxidation was minimally reduced in samples of pork and chicken thighs treated with 0.00715% (*w/w*) of CEO.

### 4.1. Lipid Oxidation in the Direct Addition of CEO

Lipid oxidation is a main cause of degradation in foods [74] because it induces modifications of muscle lipids and proteins, affecting the organoleptic and nutritional properties of meat and meat products. Oxidative reactions of lipids in muscle systems begin in the intracellular phospholipid fraction at the membrane level due to the high content of polyunsaturated fatty acids in their composition, which are the main substrates in these reactions. Furthermore, the presence of transition metals, such as iron, facilitates the generation of species capable of removing a proton from an unsaturated fatty acid, thus favouring the development of lipid oxidation [75]. This process forms compounds such as hydroperoxides and, ultimately, byproducts such as aldehydes (e.g., malondialdehyde). Salgado et al. [57] suggested that lipid oxidation can occur during post mortem handling. Lipid oxidation is measured using the thiobarbituric acid reactive substances (TBARS) or thiobarbituric acid (TBA) method, peroxide value or peroxide index (PV), and the content of free fatty acids (FFV).

The addition of CEO in meat and meat products minimises the production of malondialdehyde (MDA) during the research period. MDA is an oxidative marker that allows the identification of damage caused by lipid oxidation in food and biological systems. Phenols can act by removing MDA in food by producing stable dimers and trimers [76]. The neutralisation of oxidative compounds is due to the phenolic compounds having a hydrogen atom donor capacity, which stabilises oxidative reactions. MDA can be measured by analytical methods such as TBARS. Ghasemi et al. [22] reported a decrease of 18.53% of TBARS in frozen chicken nuggets during 90 days. Likewise, Tajik et al. [63] obtained a reduction of up to 73% compared to the control samples due to the inhibition of aldehydes and ketones generated in autooxidation. Both works agree that CEO was the best treatment compared to the other proven EOs.

It is important to consider that each matrix is different; therefore, its TBARS index varies from one to another. There are a variety of criteria concerning the limit value of rancidity and acceptability. For beef, Martins et al. [48] indicated that oxidative rancidity can be detected at values greater than 0.5 mg MDA/kg. Abdel et al. [34] reported the detection of a certain rancid flavour when the MDA content reaches 0.6 mg/kg. Uzun Özcan et al. [77] reported that the threshold limit value for rancidity is 1 mg MDA/kg, and Ugalde et al. [64] pointed out that, for meat products, the acceptable limit of lipid oxidation is 1 mg MDA/kg. Campo et al. [78] established that the maximum permissible limit in beef should be 2 mg MDA/kg. However, for Insausti et al. [79], the threshold for detecting off-odours and off-taste for humans is 5 mg MDA/kg. Xiong et al. [80], meanwhile, commented that some consumers can detect a rancid taste in pork from 0.5 mg MDA/kg onwards. Martínez et al. [81] mentioned that for fresh pork meat, a value of 1.5 mg MDA/kg is closely related to a perceptible and unacceptable off-odour. Saricaoglu and Turhan [58] expressed that the range of acceptability for chicken meat products is 1–2 mg MDA/kg since this type of product is more vulnerable to oxidation, given its high fat content and processing. Likewise, Kaban [82] maintained that, for fermented dry products, such as sucuk (dried, spiced, and fermented Turkish sausage), a certain degree of rancidity is necessary to obtain a typical and desirable aroma (slightly rotten). Whereas in fish meat, Nunes et al. [83] highlighted that the maximum limit level is 5 mg MDA/kg. Hu et al. [42] explained that fish meat structure is more prone to degradation due to its high content of polyunsaturated fatty acids, as well as pro-oxidants (metal ions) or enzymes.

The PV is employed to evaluate the degree of rancidity of the fat in the food; it is related to the measurement of the products formed at the beginning of oxidation (peroxides and hydroperoxides). This formation occurs due to the union between an oxygen molecule and unsaturated fatty acids. Oxidation depends on storage temperature. Thermal damage can release and activate metal ions, which influences the oxidation stability of meat tissues. The research of Guran et al. [40] and Ghasemi et al. [22] specified that the proposal for the use of CEO is based on its main components since the presence of antioxidants influences the decrease in the peroxide content. This is because phenolic compounds adhere to the fatty acid chains, completing their endings, in such a way that they inactivate free radicals, thus delaying the oxidative process.

The production of peroxides is slow during the initial stage of oxidation. This observation is related to the data provided by Ghasemi et al. [22], where it is shown that the highest value when treating chicken nuggets with 600 ppm of CEO occurs on day 60, then shows a decrease in peroxide content. The authors argued that this decrease is due to the auto-oxidation of hydroperoxides, generating secondary products such as aldehydes and ketones. Ghasemi et al. [22] indicated that the addition of the EO decreased the peroxide content by 22.56%, which could be related to the total phenolic content. The study by Guran et al. [40] observed values greater than 2.53 mmol O_2_/kg of fat (1.265 mEq O_2_/kg of fat) after 10 days when treating fish patties with 2.65 mL/kg of CEO, where CEO was the second most efficient oil in reducing lipid oxidation, the first being the EO of rosemary (*Rosmarinus officinalis* L.). A PV of less than 2 mmol O_2_/kg of fat is considered very good, and a PV not exceeding 5 mmol O_2_/kg of fat is considered good. Although PV in small quantities does not show sensory characteristics, a sensory evaluation is needed because peroxides are intermediate substances that produce secondary oxidation compounds.

The FFV focuses on determining the development of the hydrolytic oxidation of dietary fat. According to Guran et al. [40] and Ramezani-Fard et al. [55], the content of free fatty acids in fish products treated with CEO was lower than in the control samples (without EOs). The authors assumed that the decrease is due to the reduced effect of lipase activity. Ramezani-Fard et al. [55] fortified the fish products with omega-3. The addition of fatty acids may generate greater effects on oxidation reactions. In turn, the authors analysed cooking methods to verify the conserving efficiency of the oil on PUFA content. The study concluded that the quantity of free fatty acids was lower than in the unfortified samples, and the samples with the CEO had a lower content than their counterparts. Likewise, regardless of the cooking method, thermal treatments had no significant effect on the fatty acid composition in CEO-treated samples. Therefore, a positive effect of CEO against the hydrolytic rancidity of the samples was demonstrated.

### 4.2. Protein Oxidation in the Direct Addition of CEO

Proteins in meat products can undergo oxidation, which usually involves the breaking of disulfide bonds and other links in the protein structure. This can lead to changes in the texture, colour, and firmness of the meat, as well as affect water retention [84]. Protein oxidation can be evaluated by changes in colouration (amount of metmyoglobin) and total carbonyl content.

The impression of quality and freshness of red fibre meat is related to its colour; therefore, the discolouration is associated with the oxidation of myoglobin to metmyoglobin (MetMb). The increase in products generated by lipid oxidation accelerates the rate of myoglobin oxidation [85]. The study by Aliakbarlu et al. [35] evaluated the reduction in colour degradation by minimising the reaction rate of lipid oxidation. The results confirmed that the incorporation of 0.25% (*v/w*) of CEO showed a reduction in the discolouration rate of sheep meat, thus demonstrating the effectiveness of the oil against the colour change of red fibres.

### 4.3. Total Volatile Basic Nitrogen in the Direct Addition of CEO

Studies to determine the total volatile basic nitrogen (TVB-N) in fish products provide the total amount of volatile basic nitrogen, which begins to accumulate in tissues and deteriorate during storage. The TVB-N value determines whether deterioration has begun in frozen, dried, or salted seafood products stored for a short time or for long periods [86]. Guran et al. [40] studied the influence of different EOs on refrigerated fish patties produced from bonito fish (*Sarda sarda* Bloch, 1793), determining that at the beginning of the storage period, TVB-N levels were 11.56 mg/100 g for the control and 11.32 mg/100 g for samples treated with 2.65 mL/kg of CEO. At 10 days, the value was 13.41 mg/100 g for the treated samples; thus, CEO showed a positive effect on the product shelf life.

### 4.4. Sensory Evaluation of the Direct Addition of CEO

The direct addition of CEO to meat or meat products causes changes in sensory attributes. However, the overall acceptability of the products was good or passable, with certain exceptions. As evidenced by Aliakbarlu et al. [35], CEO significantly reduced oxymyoglobin oxidation, so there were no drastic discolouration changes in the samples, which explains the high scores for the odour. Similarly, Sharma et al. [59] stated that reducing oxygen contact through vacuum packaging can help reduce the oxidation of lipids and pigments; however, regarding the odour and flavour, the way in which the EO was applied showed undesirable results due to its nature. Abdel et al. [34] confirmed that the dosage of an EO can affect the general acceptance of the product because while the concentration was higher, the acceptance score, especially in the flavour attributes, decreased notably. In contrast, Ghasemi et al. [22] obtained good results in terms of general acceptability. They postulate that the incorporation of spices (such as garlic, pepper, cumin, paprika, and so on) helped mask the characteristic taste and smell of clove. Similarly, Guran et al. [40] reported that the smell of the fish and the other ingredients allowed the clove aroma to be disguised.

**Table 2:** Antioxidant activity obtained through the direct addition of clove essential oil to meat and meat products.

Matrix	Main Compounds in the EO	Method of Analysis	Evaluated Concentration	Storage	Results	Sensory Evaluation	Ref.
Product: beef Presentation: hamburgers	Eugenol (82.50%)	EO: NR Matrix: TBARS	250–500 mg/kg	−18 °C for 3 months	EO: NR Matrix: -MDA content below 0.5 mg/kg after 3 months of storage.	-Samples with 250 mg/kg (CEO) scored higher than 8 in taste and odour, with no significant difference between them and the control and samples with marjoram oil. -For the samples with 500 mg/kg (CEO), there were strong clove flavours, so their acceptability was low.	[34]
Product: pork and chicken thighs, boneless but with skin Presentation: ground meat	NR	EO: DPPH, ORAC and FAC Matrix: TBARS	0.00715% (*w/w*)	4 °C for 21 days (normal shelf life) and 35 °C for 48 h (accelerated shelf life).	EO: -Insignificant effect on DPPH and ORAC performance. -Antagonistic effect of cinnamon and CEO. Matrix: -The combination of EOs (clove/thyme) minimally reduced oxidation in both samples during accelerated and normal shelf life.	NR	[45]
Product: chicken breast Presentation: chicken nuggets	Eugenol (55.66%), caryophyllene (25.21%), humulene (5.32%) and δ-cadinene (5.07%)	EO: TPC, DPPH Matrix: TPC, DPPH, PV and TBARS	600 ppm	−18 °C for 3 months.	EO: -TPC = 66.01 mg GAE/g. -CEO showed the strongest inhibitory effect (95.21%). Matrix: -TPC at the end of the treatment = 389.02 mg/100 g -Antioxidant activity after 90 days = 45.53%. -Peroxide value: 22.56% reduction compared to the control samples. -TBARS index decreased by 18.53% to a final value of 1.06 mg MDA/kg at the end of the 90-day period.	-The samples with CEO had acceptable scores in all evaluated parameters (colour, odour, flavour, juiciness, texture, and acceptability). -Regarding overall acceptability, the samples received a good rating (that was lower than the control samples). There was no significant difference between the two types of samples.	[22]
Product: chicken meat with myopathies Presentation: chicken burgers	NR	EO: NR Matrix: ABTS, TPC and TBARS	0.01%	10 days of refrigeration	EO: NR Matrix: -Meat without myopathy: ABTS = 85.14 ± 4.37%, TPC = 71.88 ± 7.26 mg GAE/g and TBARS = 2.21 ± 0.67 mg MDA/kg of meat. -Meat with moderate myopathy: ABTS = 89.07 ± 5.70%, TPC = 79.23± 6.92 mg GAE/g and TBARS = 2.28 ± 0.61 mg MDA/kg of meat. -Meat with severe myopathy: ABTS = 84.27 ± 8.30%, TPC = 76.33± 2.61 mg GAE/g and TBARS = 1.88 ± 0.32 mg MDA/kg of meat.	NR	[51]
Product: beef Presentation: mortadella	Eugenol (80.67%)	EO: NR Matrix: TBARS and overall colour	0.066%	4.4 °C for 21 days	EO: NR Matrix: -No significant difference was observed between treatments. -Regarding overall colour, a significant difference was noted between treatments, but the values were below 3.0 in both cases (not detectable with the naked eye).	NR	[48]
Product: chicken meat Presentation: sausages	NR	EO: NR Matrix: TBARS, TPC and DPPH	0.25%	−18 ± 2 °C for 45 days	EO: NR Matrix: -MDA content = 0.34 mg/kg (45 days) -TPC = 757.49 mg/g.	-Although the results for samples with CEO were not as negative as the control samples, it was not the best treatment.	[60]
Product: chicken meat Presentation: sausages	NR	EO: NR Matrix: TBARS, TPC and DPPH	0.25%	4 ± 1 °C for 20 days	EO: NR Matrix: -TBARS index = 0.9 mg MDA/kg. -The samples with CEO had the highest total phenol content during the entire storage period compared to the other treatments.	-From day 5 onwards, all samples had an unattractive appearance, unpalatable flavour, a loss of texture, and reduced juiciness.	[59]
Product: red tilapia meat Presentation: Fish patties	NR	EO: NR Matrix: DPPH, FRAP, TBARS and AGL	0.1% (*w/w*)	None	EO: NR Matrix: -TBARS index values < 1 mg MDA/kg. -Regardless of the cooking method, this activity was higher in the samples not fortified with omega-3.	NR	[55]
Product: sheep meat Presentation: ground meat	NR	EO: NR Matrix: TBARS and MetMb	0.25% (*v/w*)	4 ± 1 °C for 9 days	EO: NR Matrix: Data with CEO was not as effective compared to the other treatment.	-At the end of the test time, the CEO-treated samples had good scores for colour and overall acceptability but low scores for odour.	[35]
Product: bonito fish Presentation: fish patties	NR	EO: NR Matrix: TBARS, TVB-N, PV and FFA	2.65 mL/kg	4 °C for 16 days	EO: NR Matrix: Samples with CEO were measured only up to day 10. They had the following results for TBARS, TVN-N, PV and FFA: 3.02 mg MDA/kg, 13.41 mg/100 g, 2.53 mmol O_2_/kg fat and 1.57 g oleic acid/100 g, respectively.	-The analysis revealed that in terms of colour, appearance, odour, and texture, there were no significant differences between the groups. However, the samples with CEO had low scores for taste and overall acceptability.	[40]
Product: turkey meat Presentation: hamburger	Eugenol (83.8%), eugenol acetate (5.2%), β-carhyophyllene (1.1%) and α-humulene (0.9%).	EO: NR Matrix: DPPH, ABTS, FRAP, β-caroteno and α-amilasa	1% (*w/w*)	Frozen until freeze-dried.	EO: NR Matrix: -IC_50_ of the ABTS = 5.20 mg/mL. -DPPH = 2.05 mg/mL. -β-carotene bleaching (60 min = 9.87 mg/mL. -α-amylase assays = 861.18 mg/mL. -FRAP = 34.64 uM Fe (II)/g.	NR	[47]
Product: buffalo meat Presentation: hamburgers	Eugenol (59.97%), β-caryophyllene (15.36%), 2-methoxy-4-[2-propenyl] phenyl acetate (13.21%) and α-humulene (3.93%)	EO: NR Matrix: TBARS	0.1%	8 °C for 9 days	EO: NR Matrix: -TBARS value (end of the test period) = 0.59 mg MDA/kg. -The level of oxidation was 27%, 37% and 73% lower than the samples with 0.1% and 0.2% grape seed extract and the control samples, respectively.	NR	[63]
Product: beef Presentation: minced	Eugenol (75.20%), benzyl salicylate (14.75%) and propylene glycol (6.02%)	EO: TPC, FRAP and DPPH Matrix: TBA	0.03–2% (*v/v*)	4 °C for 9 days	EO: -TPC = 635.327 mg GAE/mL -FRAP = 4.357.45 ± 28.83 mmol Trolox/mL -DPPH, IC50 = 0.14 ± 0.02 µL/mL Matrix: The results indicated that CEO delayed lipid oxidation over 9 days (0.2 mg MDA/kg).	NR	[70]
Product: beef Presentation: kavurma (traditional cooked meat product of Türkiye)	Eugenol (88.4%) and α-humulene (6.0%)	EO: NR Matrix: Peroxide number and TBARS	0.5 and 1%	4 °C for 13 weeks	EO: NR Matrix: -At the end of the treatment, the peroxide value was higher in controls K1 (5.0 ± 0.03 mEq g/kg) and K2 (5.6 ± 0.03 mEq g/kg, vacuumless) than in treated samples B (4.0 ± 0.04 mEq g/kg, 1% CEO) and C (3.6 ± 0.05 mEq g/kg, 0.5% rosemary oil + 0.5% CEO). -In the groups to which CEO was added, the TBARS value remained below the legal limit (3 mg MDA/kg) throughout the storage period. In the control groups, it exceeded this limit after the 11th week.	-At the beginning of the study, the smell of groups not containing EO received high scores from the panellists. EO groups were rated as having intense aromas by the panellists. It was stated that samples with rosemary and clove oil had a disturbingly intense smell. -It was found that the sensory quality scores of the control groups decreased at the end of the storage period.	[72]

NR: Not reported; DPPH: Capacity of antioxidants to scavenge DPPH (1,1-diphenyl-2-picrylhydrazil) radicals; ORAC: Oxygen radical absorbance capacity; FAC: Fractional antioxidant capacity; TBA: Thiobarbituric acid; TBARS: Thiobarbituric acid reactive substances; TPC: Total phenolic content; GAE: Gallic acid equivalent; MDA: malondialdehyde; ABTS: Capacity of antioxidants to scavenge ABTS (2,2′-azino-bis(3-ethylbenzothiazoline-6-sulfonate)) radical cation; FRAP: Ferric-reducing antioxidant power; MetMb: Metmyoglobin; TVB-N: Total volatile basic nitrogen; PV: peroxide value; and FFA: Free fatty acids.

## 5. Edible Films and Coatings Containing CEO

One of the main limitations in using EOs as additives is their strong aroma and prominent flavour [50]. As evidenced in the previous section (Table 2), the direct application of CEO negatively influenced certain attributes related to the sensory perception evaluated in the different matrices of some studies [34,40]. Likewise, clove is a photosensitive and thermolabile substance since it can easily decompose under normal environmental conditions [15]. In view of this problem, some authors investigated the EO through the implementation of edible films and coatings in order to minimise the adverse effects of its direct addition, thus increasing its activity and effectiveness against deterioration reactions and microbiological growth. The main objective of this barrier technology is to minimise contact between the product matrix and its environment, thereby inhibiting bacterial growth and slowing down oxidative and other degradation reactions. Regarding the quantification of studies, a total of 31 articles were obtained that analysed the EO through this form of application, of which 24 focused solely on the in vitro and in vivo antioxidant activity of the oil and some studied two activities. Biopolymers and their mixtures are widely used as a solid phase for film production in this case. Table 3 shows the studies in which the influence of CEO was analysed concerning the reduction in oxidative processes.

The incorporation of CEO undoubtedly increased the antioxidant capacity of edible films and coatings compared to control treatments. However, it is difficult to directly compare the collected studies due to the variation in the composition between matrices, as well as the concentration of CEO used. The results (Table 3) showed that the incorporation of EO increased the total phenol content (TPC) and, therefore, the antioxidant activity of the films. Dehghani et al. [36] suggested that this increase is due to the fact that at least a fraction of the EO is not strongly bound to the matrix, which allows its antioxidant properties to be maintained.

Navikaite-Snipaitiene et al. [49] evaluated the antioxidant activity of coatings containing CEO and eugenol and the effect on the quality and shelf life of beef. The comparison of the biofilms provides an understanding of the release rate of the active substances in these coatings and their influence on the free radical scavenging activity. The study concluded that the antioxidant activity in the coatings with the eugenol molecules was greater than in the biofilms with CEO because a faster release of active compounds occurred. Ugalde et al. [64] explained how the release mechanism of the active compounds from an EO in a polymeric matrix occurs, which is based on two stages. The first stage, also known as the explosion stage, consists of the rapid release of the EO. This process occurs when the polymeric network comes into contact with an aqueous medium, which generates a volumetric expansion of the polymer where the molecules are released and diffused throughout the system. In the second stage, the activity of these molecules begins to decrease since undesirable products have developed in the food matrix due to the degradation of lipids and proteins, as well as pH alterations, which lead to a slower and decreasing diffusion until it becomes null. Shukla et al. [61] explained that the exposure stage is around the first 10 to 15 days of storage time. Likewise, the authors argued that the concentration of the EO is directly proportional to the release mechanism. Therefore, the coating containing a high concentration of CEO was the only treatment in which the analysis was performed in the evaluated time. After 10 days, a decrease in antioxidant activity was evident.

### 5.1. Lipid Oxidation in Edible Films and Coatings Containing CEO

When lipids interact with oxygen molecules, deterioration reactions known as oxidative rancidity are generated. These transformations reduce the nutritional value of the food and give rise to unpleasant odours and flavours [87]. Regarding PV, the information extracted indicates that the use of coatings decreased lipid oxidation. Dehghani et al. [36] mentioned that the acceptable limit for hydroperoxide formation is 5 mEq O_2_/kg lipid. Roy et al. [56] found that the peroxide value in pork belly treated with films containing 0.75% (*w/v*) of CEO at the end of the investigation time (15 days) was lower when compared to control samples. Ansarian et al. [21], who studied the effect of CEO incorporated in basil seed gum on camel meat, assumed that the reduction in PV is due to the phenolic components of the EO since the presence of these molecules can delay the formation of peroxides, therefore decreasing lipid auto-oxidation in meat.

Nisar et al. [50] explained that the decomposition of unsaturated fatty acids leads to the formation of peroxides. These molecules indirectly influence the sensory characteristics of meat (flavour changes) as well as the loss of nutrients. The observation of PV reduction (control: 5.02, 1% CEO: 3.24 and 1.5% CEO: 2.64 mEq of O_2_/kg fat) could be associated with the CEO antioxidant activity because phenolic compounds act as potent scavengers of free radicals by donating a hydrogen atom from their OH group [50]. Similarly, El-Saber Batiha et al. [15] reported that the resulting phenolic radicals are stable molecules that do not establish or increase oxidation because they present resonant structures thanks to the phenolic ring.

When peroxide decomposes, secondary oxidation occurs and aldehydes and ketones are formed. The main aldehyde produced by this reaction is MDA [42]. Ansarian et al. [21] and Venkatachalam et al. [65] mentioned that these molecules generate unpleasant odours in meat. Therefore, the TBARS index is used to quantify the secondary products generated by auto-oxidation, thus providing information on the progress of the product oxidation. Nisar et al. [50] evaluated the effectiveness of a film containing CEO as an antioxidant in samples of Wuchang bream (*Megalobrama ambycephala*), where a decrease in the amount of MDA was evident in the treated samples compared to the control samples (control: 3.01, 1% CEO: 1.70 and 1.5% CEO: 1.26 mg MDA/kg of tissues). The authors assumed that this reduction is due to the bioactive molecules present in the EO, given their antioxidant potential, which allowed the elimination of free radicals present in their samples, thus hindering lipid oxidation. However, Jalali et al. [44] stated that phenolic compounds do not absorb oxygen but rather prevent the formation of free radicals, thus neutralising fatty acids. Likewise, some authors, including Xiong et al. [68], suggested that the limitation of the formation of secondary compounds is due to the barrier effect of films and coatings, which prevents direct contact between oxygen and meat.

Bogdanović et al. [88], studying *Sardine pilchardus* and *Boops boops*, suggest that to guarantee the quality of fish, the maximum acceptable level is 5 to 8 mg MDA/kg. However, Xiong, et al. [68] argued that in fish meat with a MDA content greater than 2 mg/kg, there are already certain undesirable odours, but it does not affect the quality of the product.

A number of authors agree that the main compounds of CEO can inhibit the oxidation reactions in a food item. Navikaite-Snipaitiene et al. [49] studied the release of eugenol and its influence on oxidative rancidity. The study concluded that eugenol is responsible for the inhibition of lipid oxidation.

On the other hand, Shukla et al. [61] analysed the relationship between the concentration of CEO in coatings and storage time with respect to the decrease in MDA content present in meat. The results of that study confirmed that, as time passed, coatings with a higher concentration of the EO prevented the increase in lipid rancidity more efficiently because the conversion of hydroperoxides into aldehydes and other secondary oxidation products was slower, so the TBARS index remained within the maximum acceptance limit. Edible films and coatings based on biopolymers are effective because they act as a barrier between the food matrix and the environment, limiting contact between oxygen and the meat and, thus, hindering chain reactions of lipid oxidation. The work carried out by Jalali et al. [44] and Venkatachalam and Lekjing [65] showed that the FFA content increased gradually from 0.22 to 4.44% for silver carp and from 0.67 to 3.17% in pork patties. However, despite this increase, the results in the samples with coatings containing CEO were significantly lower compared to the control samples.

### 5.2. Protein Oxidation in Edible Films and Coatings Containing CEO

Protein oxidation can result in the formation of reactive compounds that negatively affect the flavour and sensory quality of a food product. Protein oxidation is related to a decrease in sulfhydryl or thiol groups, which undergo condensation reactions, thus obtaining disulfide compounds [89]. Susceptibility to changes due to discolouration is related to heme protein levels since myoglobin can act as a pro-oxidant agent in meat [21]. In general, the application of edible films and coatings delayed the discolouration of meat to a certain extent. Venkatachalam and Lekjing [65] stated that colour increased in pork meat (patties) treated with 6,400 μg/mL of CEO incorporated in chitosan and nisin. Meanwhile in fish meat, Xiong et al. [68] and Nisar et al. [50] observed yellow colour changes as the days passed. However, compared to the control samples, an improvement was observed. The authors presumed that stability is due to the antioxidant capacity of CEO since it delays lipid oxidation, so indirect oxidation by proteins decreases.

Like lipids, proteins are susceptible to ROS (superoxide and hydroxyl radicals, peroxides and hydroperoxides). Ansarian et al. [21] explained that indirect protein oxidation can be generated due to the formation of aldehydes and ketones (oxidation products of peroxides and hydroperoxides) since these molecules can bind to amino acid side chains, such as arginine, lysine, and threonine, through covalent bonds or to amino acid residues like proline, generating carbonyl compounds. Wie et al. [71] and Ansarian et al. [21] observed that in donkey meat and camel meat, respectively, the use of coatings with CEO had a significant effect on the increase in the total carbonyl content with respect to the control samples thanks to the barrier effect of the edible films and coatings.

### 5.3. Total Volatile Basic Nitrogen in Edible Films and Coatings Containing CEO

A number of researchers have evaluated the influence of coatings with CEO against the deterioration of muscle tissues using the TVB-N index, which quantifies the presence of amino group compounds and ammonia. Shukla et al. [61] studied the effect of CEO incorporated into chitosan in chicken meat with concentrations of 0.25, 0.5, 0.75, and 1% and stored at 4 °C for 35 days. They attributed the increase in the concentration of volatile basic nitrogen to protein degradation since volatile bases and other nitrogen compounds were produced. Hosseini et al. [41] mentioned that this index is observed mainly in fish and chicken meat. White meat has a lower fat content, so proteins are the main compounds that suffer deterioration, generating volatile bases or amines. The increase in these substances is due to proteolysis induced by enzymatic activity or by the presence of bacteria. For this reason, their research was based on comparing the influence of clove and lemon verbena EO on chicken meat. They found that CEO caused a significant decrease in nitrogen compared to lemon verbena EO. The authors assume that this result is due to the main compounds found in cloves, especially eugenol molecules.

Nisar et al. [50] evaluated the influence of pectin coatings enriched with 1 and 1.5% (*w/v*) CEO on fillets of sea bream fish. The study showed that TVB-N values of the samples treated with 1% CEO (27.40 mgN/100 g) and 1.5% CEO (23.50 mgN/100 g) at 15 days were lower than the control (42.76 mgN/100 g). The European Commission declared that the maximum acceptance limit for volatile basic nitrogen for marketing fish is 35 mg N/100 g. In contrast, Dehghani et al. [36] indicated that, for sweet fish, the limit value is 25 mg N/100 g; in turn, Giménez et al. [90] mentioned that when a sample reaches this value, the beginning of deterioration can be observed.

### 5.4. Sensory Evaluation of Edible Films and Coatings Containing CEO

When meat is stored, its structure begins to suffer a series of deterioration reactions, such as oxidation of lipids and proteins and colour variations (oxidation of myoglobin). These changes can be evaluated through a sensory evaluation, where the changes in quality are observed as time passes [91]. Through the information extracted, i.e., the sensory analyses carried out in the different studies, it was observed that all the samples covered in the coatings with CEO had a better acceptance with respect to their counter samples. However, most of the products did not manage to pass the evaluation in the established research time due to the evident decrease in the sensory attributes, mainly in smell and texture [42]. In the case of the products that passed the sensory evaluation, the results revealed that they did not reach an acceptable threshold, obtaining values less than 6 on hedonic scales [52].

Ansarian et al. [21] argued that low sensory scores are due to the oxidation of lipids and proteins, which triggers the production of oxidation compounds such as aldehydes, ketones, alcohols and ammonia that are responsible for causing putrid odours, slime formation and discolouration in the meat. The authors reported that the concentration of CEO is a key factor in reducing product deterioration for both shelf life and acceptance. Shukla et al. [61] showed that samples with concentrations of less than 1% had greater acceptability and higher scores, but their shelf life was reduced. Nisar et al. [50] demonstrated acceptable effects for their samples of gilthead seabream (*Megalobrama ambycephala*) since the EO masked the fishy odour and reduced discolouration. Although its final score was 4, it was considered passable for this type of product. The analysis by Stoleru et al. [62] did not reveal any organoleptic migration of the EO, although CEO was added to the film along with other EOs, so they assumed that this combination masked the aroma and flavour of the EO in question.

**Table 3:** The antioxidant activity of clove essential oil incorporated into edible films and coatings for meat and meat products.

Matrix	Main Compounds of CEO	Method of Analysis	Tested Concentration	Storage	Incorporation Material	Results	Sensory Evaluation	Ref.
Product: pork belly Presentation: pieces	NR	EO: DPPH and ABTS Matrix: PV	0.75% (*w/v*) clove essential oil in pickering emulsion with cellulose nanofiber	10 °C for 15 days	Gelatin/agar (1:1 w:w) with glycerol as plasticiser (30% *w/w*)	EO: -Elimination of free radicals, reaching approximately 31% for DPPH and up to 61% for ABTS. Matrix: -Peroxide value (after 15 days) = 16 mEq/kg compared to the samples without the film (22 mEq/kg). -Extension of shelf life was not analysed.	NR	[56]
Product: camel meat Presentation: ground	Eugenol (76.07%), caryophyllene (8.72%), aceteugenol (5.17%) and α-humulenen (4.95%)	EO: TPC, DPPH and ABTS Matrix: PV, TBARS and Total carbonyl	2.5, 5 and 10 mg/mL	4 °C for 20 days	Basil seed gum (1% *w/v*) with glycerol as plasticiser	EO: -The in vitro study revealed that the addition of CEO caused a significant increase in the phenolic content of the film (19.26–45.21 mg GAE/g). -Free radical scavenging capacity reached values of 30.66–81.03% and 34.77–82.49% for DPPH and ABTS, respectively. Matrix: -Peroxide value = 4.03 mEq/kg of lipids (20 days). -TBARS index = 1.03 mg MDA/kg (20 days). -Total carbonyls = 0.84 nmol/mg of protein (20 days). -Extension of shelf life was not analysed.	-The camel meat during 10 days of storage period received a score of 5.4, indicating that it did not reach an acceptable threshold.	[21]
Product: beef Presentation: pieces	Eugenol (85.7%), eugenol acetate (7.9%) and β-caryophyllene (4.5%)	EO: DPPH and ABTS Matrix: NR	150 μL in 20 mL of chitosan solution	7 °C for 2 days.	Poly(lactic acid) films immersed in oil-loaded chitosan solution (1.3 % *w/v*)	EO: -CEO increased the elimination of radicals from the chitosan coating (43%), due to its main compound, eugenol. Matrix: NR -Extension of shelf life was not analysed.	-The analysis did not reveal any organoleptic migration of the EO.	[62]
Product: Pacific mackerel (*Pneumatophorus japonicus*) Presentation: fillets	NR	EO: NR Matrix: TVB-N and TBARS	2–3% (*w/w*)	4 °C for 12 days.	Collagen (0.6% *w/v*), carboxymethyl cellulose (0.3% *w/v*).	EO: NR Matrix: -The mackerel fillets treated with CEO films showed a TVB-N content of 20.61 and 16.28 mg/100 g. -TBARS value: the MDA content reached the maximum permissible limit on day 8. -The pH showed only a slight variation compared to the control sample. -The application of combined EOs and collagen on Pacific mackerel efficiently maintains quality and extends shelf life.	-Regardless of the treatments, no sample was able to pass the sensory analysis due to the putrid odour emitted by the fillets. -The texture had deteriorated and the fillets’ appearance showed yellow pigmentation. -The samples with the highest concentration of CEO received an acceptable score until day 9.	[42]
Product: Atlantic salmon (*Salmo salar*) Presentation: pieces	NR	EO: NR Matrix: TBARS and DTNB	0.5% (*w/w*)	4 °C for 15 days.	Salmon bone gelatine (2% *w/v*), chitosan (2% *w/v*), clove oil (0.5% *v/v*), and soy lecithine powder (0.5% *w/v*)	EO: NR Matrix: -During the testing period, the TBARS value in the samples with CEO coatings remained below 1 mg MDA/kg. -The free thiol group value was 53.85 nmol thiol/mg on day 0 and decreased during storage to values of 28 to 40 nmol thiol/mg at 15 days. -The shelf life of the samples was extended by 5 days.	NR	[68]
Product: chicken breast Presentation: pieces	Eugenol (79.4%), β-caryophyllene (13.36%), eugenol acetate (4.49%) and α-caryophyllene (1.67%).	EO: NR Matrix: TBARS and TVB-N	0.2 and 0.5% (*w/v* of coating solution)	4 °C for 15 days.	Sodium alginate (2% *w/v*), calcilum chloride (2% *w/v*)	EO: NR Matrix: -Lipid oxidation increased rapidly after 5 days of storage; minimal TBA values (0.54 mg MDA/kg) were recorded in the Le_0.5_C_0.5M_ sample (0.5% lemon essential oil and 0.5% CEO in a modified atmosphere). -Higher CEO concentrations had no significant effect on any of the variables, neither under normal packaging conditions nor in a modified atmosphere. -TVN (total volatile nitrogen) decreased from day 0 to day 5 and then steadily increased. In Le_0.5_C_0.5M_, this variable remained constant from day 0 to day 5 and then increased. On day 15, the TVN level was 36.66 mg/100 g, while in the control sample, it was 57.33 mg/100 g. -Coating chicken breast with sodium alginate incorporated by essential oil, as well as modified atmosphere packaging, successfully increased the shelf life of chicken.	-This evaluation revealed that as the concentration of CEO increased, the scores for the attributes decreased.	[41]
Product: chicken Presentation: burgers	NR	EO: DPPH Matrix: TBARS and TVB-N	0.25, 0.5, 0.75 and 1%.	4 ± 1 °C for 35 days	Chitosan (0.5%, 1%, 1.5%, and 2.0%)	EO: -After the first 15 days of storage, antioxidant activity decreased in all coatings. -The treatment with the highest concentration of CEO was the only one that maintained antioxidant activity throughout the research period, with a final value of 15.11% (on day 15). Matrix: -The film containing the highest concentration of CEO was the only treatment that passed its analysis within the established time, making it the best treatment. -The TBARS index was 0.98 ± 0.03 mg MDA/kg. -The TVB-N value was 22.80 ± 0.16 mg/100 g. -The shelf-life was enhanced by 5 days by the edible coating of chitosan alone (T 3 ), but the shelf-life was enhanced by 20 days with an edible coating of chitosan and clove essential oil as compared to the control.	-The edible coating with 0.5% CEO received higher sensory scores because at this concentration, the edible coating did not inhibit the sensory attributes of the burgers. Coatings with concentrations higher than this reduced the flavour and aftertaste of the samples.	[61]
Product: pork Presentation: patties	Eugenol (75%)	EO: NR Matrix: FFA, PV, TBARS and MetMb	6400 μg/mL (of film solution)	4±2 °C for 15 days	Chitosan (2 % *w/v*), glycerol (0.5 mL/g Chitosan) and nisin	EO: NR Matrix: -In the pork patties with CEO edible films, the MetMb content (54.10 to 63.36%), FFA (0.67 to 3.17%), PV (0.80 to 3.67 mEq/100 g), and TBARS (0.69 to 3.27 mg MDA/kg) gradually increased during storage.the	-Regardless of the treatments, the samples presented unpleasant odours; however, the presence of clove aroma was evaluated and received acceptable scores. -They also showed discolouration. A minimum acceptability threshold of 3 was established, and the control remained acceptable for 12 days while treatment with CS-CO extended shelf life by at least 5 more days.	[65]
Product: Beef Presentation: Slices of sucuk (traditional Turkish fermented sausage)	Cymol (26.29%), α-pinene (20.65%), eugenol (17.02%) and 3-carene (11.62%)	EO: NR Matrix: TBARS and MetMb	1.50% (*v/v* of coating solution)	4°C for 45 days	Deboned chicken meat protein (4% *w/v*), glycerol (40% *w/w* of protein)	EO: NR Matrix: -The application of the CEO coating reduced the weight loss of the samples, delayed colour deterioration, and improved the storage quality of the thermally treated sucuk. -TBARS acceptable value was 2.00 mg MDA/kg; the control treatment reached this value on day 30, while the CEO15 treatment extended the time to 45 days.	NR	[58]
Product: Wuchang bream (*Megalobrama ambycephala*) Presentation: fillets	NR	EO: NR Matrix: PV, TBARS, TVB-N and MetMb	1% and 1.5% (*w/v* of film solution)	4 °C for 15 days	Pectin (3.5% *w/v*) and glycerol (25% *w/w* of pectin)	EO: NR Matrix: -The values obtained from the VP (control: 5.02, 1% CEO: 3.24 and 1.5% CEO: 2.64 mEq of O_2_/kg fat), TBARS (control: 3.01, 1% CEO: 1.70 and 1.5% CEO: 1.26 mg MDA/kg of tissues) and TVB-N (control: 42.76, 1% CEO: 27.40 and 1.5% CEO: 23.50 mg N/100 g) analyses at the end of the 15 days were lower compared to the control samples representing a 3-day extension of shelf life.	-The results of the organoleptic analysis showed that the films with CEO had no negative effects on the acceptability. The fillets with this covering showed a firmer texture, less of a fishy odour, and a stable colour when compared to the control treatment. -The score for the sensory parameters decreased as storage time increased; the panellists’ final score was 4. However, for the fish samples, this evaluation could be considered acceptable.	[50]
Product: tambaqui (*Colossoma macropomum*) Presentation: fillets	NR	EO: NR Matrix: TBARS	0.08 and 0.16% (*w/v* of coating solution)	−18 ± 1 °C for 120 days	Chitosan (2% *w/v*) and lactic acid (1% *w/v*)	EO: NR Matrix: -MDA content in the samples with CEO films (0.16% and 0.08%) was 0.75 and 0.80 mg/kg at 120 days, respectively.	-The score for the CEO films (0.08%) was 5.4, while the scores for the CEO films (0.16%) reached 4.7. These scores both correspond to “neither like nor dislike”.	[66]
Product: grass carp (*Ctenopharyngodon idellus*) Presentation: pieces	Eugenol (44.9%) and caryophyllene (26.5%)	EO: NR Matrix: Enzymatic activity	0.1, 0.5 and 1% (*w/v* of coating solution)	4 °C for 15 days	Chitosan (2% *w/v*) and glycerol (0.5% *w/v*)	EO: NR Matrix: -It was evidenced that as the concentration of CEO in the films increased, the activity of cathepsins B and B+L increased, reaching an inhibition of 65% by day 11. -The coatings had an inhibitory effect on the activity of cathepsin D only up to a certain point, as it had decreased by only 7% by day 11. -The coatings prevented proteolysis and the deterioration of the texture of the refrigerated fillets (shear force). -Extension of shelf life was not analysed.	NR	[69]
Product: beef Presentation: fillets	Eugenol (67.6%), aceteugenol (16.8%) and trans-caryophyllene (10.8%)	EO: DPPH Matrix: TBARS	12.5% CEO (*w/v* of emulsion) and 12.5% eugenol (*w/v* of emulsion).	4 ± 1 °C for 14 days	a. Emulsion of hydrophobically modified waxy maize starch (25% *w/v*) with CEO. Emulsions (8 to 34.8 g) were mixed with acrylic component (92 to 65.2 g). The final CEO concentration ranged from 2.5 to 20% by weight. b. Dry coating of cellulose acetate (77%, 87%, and 93% weight) in acetone with CEO (7%, 13%, and 23% weight).	EO: -The biofilms with CEO and eugenol exhibited free radical inhibition by 87–92% and 90–94%, respectively. Matrix: -The changes in colour were not significant, meaning that the red colour of the meat was maintained in the packages with antioxidants. -Lipid oxidation was 0.966 mg MDA/kg after 14 days. -Compared with the control, AC/S/EU1, AC/S/EU2, and CA/EU treatments maintained the acceptability limit below 2 mg MDA/kg for an additional 7 days.	NR	[49]
Product: bluefin tuna (*Thunnus thynnys*) Presentation: fillets	NR	EO: NR Matrix: TVB-N and TBARS	0.5% (*v/v* of film solution)	2 ± 1 °C for 15 days	Soy protein (5% *w/v*), montmorillonite (0 to 0.5% *w/v*) and glycerol (1.25% *w/v*)	EO: NR Matrix: -At the end of the testing period, the samples with films maintained values of 33 mg TVB-N/100 g, unlike the control samples, which reached values of 30 mg TVB-N/100 g during the first week. -The MDA content of the control increased its initial value (0.18 mg MAD/kg) to 1.8 mg MAD/kg at the end of storage, while the film treatments were significantly lower. This could be considered an extension of the product’s shelf life.	NR	[37]
Product: rainbow trout (*Oncorhynchus mykiss*) Presentation: fillets	Eugenol (77.57%), eugenol acetate (10.23%) and caryophyllene (7.51%)	EO: DPPH Matrix: TVB-N, PV and TBARS	0.5, 1 and 2%	4 ± 1 °C for 16 days	Farsi gum (2.5% *w/v*), glycerol (0.75% *v/v*)	EO: -Increased radical scavenging activity (93.7%) compared to thyme EO (53.5%) was observed. Matrix: -Taking into account the limit of the TVB-N index, the shelf life of controls and EO-loaded coated samples was 8 and more than 12 days, respectively. -The lowest amount of PV was measured in the coated fillets containing a 2% CEO + SEO coating formulation. -The amounts of TBARS were below the maximum limit during 16 days of storage. -At the end of storage, the lowest amount (1.7 mg MDA eq./kg) of TBARS was measured in coated fillets containing 2% CEO + SEO.	-The coating with the lowest concentration of CEO received the highest score. However, the analysis was concluded on day 8 as all samples exhibited very low scores.	[36]
Product: sutchi catfish (*Pangasius hypophthalmus*) Presentation: fillets	Eugenol (78.95%), caryophyllene (4.26%) and benzene, 1-ethyl-3-nitro (2.46%).	EO: TPC and DPPH Matrix: TBARS and PV	0.25% (*w/v*) at 1:1 ratio (fish/dip solution)	0–2 °C for 15 days	Polyethylene pouches. Dip solution: EO in ethanol (1:2) and diluted to a final concentration of 0.25% (*w/v*)	EO: -The CEO showed a higher phenolic content, and it increased the antioxidant activity of the film by 83% compared to the control treatment. Matrix: -At the end of the research period, the samples reached an average of 0.57 mg MDA/kg, indicating that the film was effective in delaying lipid oxidation. -Similarly, the samples remained below the fat acidity limit (3% oleic acid). -The PV reached the maximum permissible limit by day 9. -EO-treated samples showed an extension of shelf life for PV (from 9 to 18 days) and TBARS (from 13 to 18 days).	-The samples with CEO coatings exhibited greater chewiness and gumminess. However, the analysis was only conducted up until day 11.	[23]
Product: pork Presentation: sausages	Eugenol (85%)	EO: DPPH Matrix: TBARS	1.5% (*v/v*)	2 ± 2 °C for 15 days.	Corn starch (3% *w/v*), glycerol (30% *w/w*)	EO: -The antioxidant activity of the CEO in the film was 53% at the end of its storage period. Matrix: -The samples treated with active films remained below the permissible limit (1 mg of MDA/kg) throughout the storage period. -Extension of shelf life was not analysed.	-It was evident that the colour showed no significant difference between the control samples and the treated ones. -However, the flavour and odour did have a significant effect.	[64]
Product: pork Presentation: sausages	Eugenol (75%)	EO: NR Matrix: PV and TBARS	1.5% (*w/v* of chitosan solution)	4 ± 2 °C for 25 days	Chitosan (2% *w/v*)	EO: NR Matrix: -Regarding the samples with CEO coatings, both the PV and the TBARS index were lower than the data from the other samples. -No limit values of PV and TBARS were described that would allow for considering an extension of shelf life.	-The researchers observed decreasing acceptance as the storage period progressed. -The attributes with the lowest scores were smell and taste. -The overall acceptability of the samples was below 5 on day 20.	[46]
Product: silver carp Presentation: fillets	Eugenol (59.29%), propilenglicol (11.29%), benzotiofeno-3-carbonitril, 4,5,6,7-tetrahidro-2-(3-etoxi-4-hidroxibencilidenamino) (9.67%) and β-caryophyllene (5.01%)	EO: NR Matrix: PV, TVB-N and FFA	1 and 1.5% (*v/v*)	4 °C for 16 days	Alginate (1.5% *w/v*), carboxyl methyl cellulose (3% *w/v*	EO: NR Matrix: -The values of the coatings with the highest concentration (1.5%) remained below the limit (34.35 mg N/100 g) until the end of the trial period. -An extension of shelf life from day 7 to day 10 could be recognised from the TVB-N value. -At day 16, the PV in the coated samples (between 4.5 and 5 mEq/kg) was lower than in the control (between 6 and 6.5 mEq/kg). -The FFA of the fresh fish was approximately 0.22% oleic acid. At the end of the storage period, the samples coated with 1.5% of CEO reached a lower FFA value of 2.86% oleic acid in comparison with the samples coated with 1% of CEO (3.05%) and the control (4.44%).	-Scores below 4 were considered unacceptable. The samples with 1% and 1.5% CEO coatings reached these scores on days 12 and 16, respectively.	[44]
Product: beef Presentation: fillets	Eugenol (83.3%) and caryophyllene (10.6%)	EO: NR Matrix: TBARS	0.5 to 4% (*v/v*)	4 °C for 15 days	Corn starch (6% *w/v*), glycerol (50% *w/w* of CS), xanthan gum (0.1% *w/v*), and cooking oil (2% *v/v*)	EO: NR Matrix: -The TBARS index for the samples with CEO was lower than that of the control samples (without CEO) at the end of the research period, with a value of 1.32 mg MDA/kg. -Redness values in the samples decreased significantly. -Extension of shelf life was not analysed.	-The samples coated with films containing CEO had a distinctive but pleasant taste and smell on the first day. -However, the scores on the hedonic scale dropped to the rejection limit (6 points) by the 12th day of the analysis.	[52]
Product: sardine (*Sardina pilchardus*) Presentation: fish patties	NR	EO: ABTS, FRAP and PCL Matrix: TBARS and TVB-N	0.75 mL/g of SPC	2 ± 1 °C 13 days	Sunflower protein (5% *w/v*) and glycerol (1.5% *w/v*)	EO: -In the films with CEO, the increase in antioxidant capacity was notable in all assays. -This activity was greater against the ABTS radical, unlike the superoxide anion radical (PCL), where its values were lower. Matrix: -In the patties that had films with CEO, the rate of MDA production was lower, especially on day 3, which allowed the values to remain low throughout the entire period. -For TVB-N, there was no significant difference between the samples. At the end of the evaluation period, the values were below 35 mg TVB-N/100 g. -Extension of shelf life was not analysed.	NR	[57]
Product: cod (*Gadus morhua*) Presentation: fillets	NR	EO: NR Matrix: TVB-N	0.75 mL/g	2 ± 1 °C for 11 days	-Gelatin (8% *w/v*). -Gelatin (6% *w/v*), chitosan (2% *w/v*)	EO: NR Matrix: -The control samples exceeded the value of 30 mg TVB-N on day 3, while the samples with films reached this value on day 9. -During fish storage, the clove film delayed or even prevented the occurrence of total volatile nitrogen. Therefore, films incorporating clove essential oil could assure an extended shelf life for chill-stored fish.	NR	[39]
Product: Buffalo meat Presentation: fillets	NR	EO: NR Matrix: TBARS	0.1% (*v/v*)	4 ± 1 °C for 12 days	Low density polyethylene pouches. Dip solutions: -Lactic acid (2% *v/v*) -Lactic acid (2% *v/v*)—CEO (0.1% the *v/v*) -Lactic acid (2% *v/v*)—CEO (0.1% *v/v*)—vitamin C (0.5% *w/v*)	EO: NR Matrix: -The immersion of buffalo meat fillets did not have the expected effect on the decrease of pH in the established study time. -The TBARS index did not have a significant reduction. However, the values for both parameters were lower than their control samples. -Use of either LA + clove or LA + clove + Vit C significantly extended buffalo meat display life at 4 ± 1 °C	-The buffalo meat with CEO coatings did not show any discolouration during the storage period (as confirmed by colour readings). -The odour scores were acceptable, although a characteristic clove smell was evident on the first day. -As the days passed, the odour diminished, likely due to the inclusion of lactic acid, which masked the aroma.	[25]
Product: pork Presentation: Chinese bacon (preserved meat product)	NR	EO: Superoxide radical scavenging activity and hydroxyl radical scavenging activity Matrix: Superoxide radical scavenging activity and hydroxyl radical scavenging activity	0.05, 0.1 and 0.2% *w/w* for CEO; 0.5, 1 and 2% *w/w* for CEO/β-CD-MOF	3 days under refrigeration (storage period) and a fermentation period of 15 days (20 °C for 5 days and from 20 to 40 °C at a rate of 20 °C per day)	CEO encapsulated in β-cyclodextrin (1:10 *w/w*) and β-cyclodextrin metal organic frameworks (1:10 *w/w*)	EO: -Superoxide radical scavenging activity of CEO = 0.610 ± 0.012 mg/mL. -Hydroxyl radical scavenging activity = 1.638 ± 0.047x10e5 mg/mL. Matrix: -The scavenging activity of CEO was significantly improved by β-CD-MOFs inclusion. -The order of superoxide radical scavenging activity was CEO/β-CD-MOFs > Vc ≥ CEO/β-CD ≥ CEO ≥ BHT > PG. The EC_50_ results showed that hydroxyl radical scavenging activities declined in the trend of BHT > CEO/β-CD-MOFs > CEO/β-CD ≥ CEO ≥ PG > Vc. -Extension of shelf life was not analysed.	NR	[67]
Product: fish (*Lateolabrax japunicus*) Presentation: fillets	NR	EO: coaxial electrospinning films (PCL) containing CEO were evaluated by ABTS and DPPH Matrix: TBARS and TVB-N	1% and 2% (*v/v*) CEO-PCL solution	Fillets stored in experimental preservation boxes at 4 °C	Polycaprolactone (18% *w/v*)	EO: -The addition of CEO doubled the antioxidant activity of the films. Matrix: -Fish fillets treated with the pads exhibited minimal fluctuations in TBA values, effectively impeding the oxidation of fats in the fish fillets. -The rate of TVB-N increase in the fish fillets subjected to the pad treatment marginally lagged behind that of the control group. -The use of composite preservation pads infused with EO significantly extended the quality guarantee period of the fish fillets from 5 days to 9 days.	-The group that included preservatives maintained sensory evaluation scores well above 3 points at 15 d, indicating the exceptional preservation performance of the prepared films in maintaining the freshness of the fish fillet	[43]
Product: donkey meat Presentation: pieces	NR	EO: NR Matrix: TBARS and carbonyl content	0.5%	4 °C for 12 days	-Nanoemulsion and coarse emulsion: WPI (1.5% *w/w*), lecithin (0.05% *w/w*), ε-polylysine (0.125% *w/w*), and clove essential oil (0.50%). -Coating solution: Carboxymethyl celulose (3% *w/v*)	EO: NR Matrix: -The combination of CEO and ε-PL effectively inhibited fat oxidation. At the end of the treatment, the TBARS index was 6.1 mg MDA/kg for the control and 3.5–3.6 mg MDA/kg for CEO-ε-PL-CMC (2.25% CMC+0.5% CEO+0.125% ε-PL). -The carbonyl content of the control was higher than that of the groups containing CEO during the storage period. At the end of the treatment, the carbonyl content was 5.8 mmol/mg for the control and between 4.8 and 5.4 mmol/mg for the samples with CEO, although there was no significant difference amongst the samples containing only CMC and CEO. -Nanoemulsion could effectively extend shelf life of refrigerated donkey meat and improve the quality of donkey meat.	-The TPA results showed that the essential oils could effectively slow down the loss of texture indexes of donkey meat and reduce protein degradation and oxidation.	[71]

NR: Not reported; DPPH: Capacity of antioxidants to scavenge DPPH (1,1-diphenyl-2-picrylhydrazil) radicals; ORAC: Oxygen radical absorbance capacity; FAC: Fractional antioxidant capacity; TBARS: Thiobarbituric acid reactive substances; DTNB: Determining the presence of free thiol group in the samples using Ellman’s reagent DTNB (5,5′-dithiobis(2-nitribenzoic acid); TPC: Total phenolic content; GAE: Gallic acid equivalent; MDA: malondialdehyde; ABTS: Capacity of antioxidants to scavenge ABTS (2,2′-azino-bis(3-ethylbenzothiazoline-6-sulfonate)) radical cation; FRAP: Ferric reducing antioxidant power; MetMb: Metmyoglobin; TVB-N: Total volatile basic nitrogen; TVN: Total volatile nitrogen; PV: peroxide value; FFA: Free fatty acids; PS/EVOH/PE: poliestireno/alcohol vinílico de etileno/polietileno; PCL: Policaprolactona; β-CD: β-cyclodextrins; β-CD-MOFs: beta cyclodextrin metal organic frameworks; PG: propyl gallate; VC: vitamin C; CMC: carboxymethyl chitosan; and ε-PL: ε-polylysine.

## 6. Encapsulated Clove Essential Oil

Applications of CEO are limited due to its low solubility (hydrophobicity), high volatility, and instability, depending on parameters such as light, temperature, and air (oxygen) [67]. In addition, it has limitations in terms of taste and smell, even when added directly to edible films and coatings as mentioned in the previous sections. Faced with this challenge, some authors, including Wang et al. [67], Gasti et al. [38], Radünz et al. [53], Wang et al. [92], and Rajaei et al. [54], have explored alternatives such as the encapsulation of bioactive compounds. This proposal seeks to provide greater stability for the bioactive compounds as well as a controlled release, thus allowing them to work with solutions or incorporating them directly into the meat. However, the main challenge is to find the ideal coating material to increase the biological activity of the EO. One of the main applications of CEO encapsulation studies is to measure and test the efficiency of the capsules in terms of reducing oxidation. Table 4 displays the information extracted regarding antioxidant activity, divided into two sections: direct addition and edible films and coatings.

In the oxidation process, the hydroxyl radical is the main ROS since it is obtained from the partial reduction of molecular oxygen. As a result of the decomposition of these molecules, lipid peroxidation and damage to their structure are generated [93]. These radicals are highly active in biological systems; therefore, it is necessary to inhibit their activity [67]. For this reason, the DPPH method is used to examine reducing compounds [53]. Gasti et al. [38] examined the ability of films with nanoparticles of CEO to eliminate free radicals. The study suggests that the antioxidant activity depends on the dose at which the nanocomposites are added to the film. The authors explained that DPPH free radicals interact with the amino groups of chitosan, which generate stable radicals, increasing the elimination of said compounds by 75%, 80%, and 85% in the bioactive films with the EO encapsulated at 1%, 3, and 5%, respectively.

In turn, Radünz et al. [53] sought to analyse the antioxidant potential of CEO compared to its nanoparticles. To do this, they analysed the antioxidant activity of the particles in different emulsifiers. The results showed that sodium alginate has a good compatibility with the EO, so the molecules with it showed greater antioxidant activity.

Another study that compared the antioxidant activity of free and encapsulated CEO was by Rajaei et al. [54], where nanogel was used as a coating material. The results revealed that encapsulation caused a significant increase in the antioxidant activity of the EO. After three hours, values of 100% were obtained in the free radical elimination activity. The authors assumed that there was a synergistic effect between the compounds, which positively influenced the interactions of the encapsulated molecules. It is necessary to understand that the coating material influences the quality and stability of the encapsulated particles because the compatibility of the encapsulant allows protection from oxidation reactions, solubility, or unwanted release [94].

Wang et al. [67] stated that the superoxide anion scavenging capacity also showed a similar trend to the hydroxyl radical scavenging activity, i.e., as the concentration of the active substances increased, the radical scavenging activity also increased. The results showed a significant improvement in the films containing CEO nanoparticles, even up to two times more than synthetic antioxidants. The application of at least two test methods is because a single test does not fully describe the free radical scavenging potential of an antioxidant since each one is based on different techniques to measure the sensitivity and reaction towards reducing molecules.

### Lipid Oxidation of Encapsulated CEO

The influence of CEO encapsulation on lipid oxidation was analysed; however, the studies did not evaluate the sensory evaluation. The research by Wang et al. [67] showed a gradual increase in TBARS as the study time passed, since there was a secondary oxidation of the lipids either due to the change in temperature or the presence of oxygen in the experimental environment.

In general, the MDA content was lower in the samples containing the microcapsules of CEO compared to the control samples, and none of them exceeded the maximum limit of MDA allowed in the People’s Republic of China (2.5 mg MDA/kg). However, the MDA content in the 2% concentration was significantly lower than in the other concentrations. For this reason, the authors confirmed that the higher the concentration of the encapsulated substances, the lower the lipid oxidation.

Wang et al. [67] evaluated the degree of oxidation of primary lipids, where a similar behaviour was observed in all samples with respect to the gradual increase in the oxidation rate. The authors suggested that this increase is due to the high temperatures in the fermentation process, and they assumed that the production of peroxides was accelerated. Hu et al. [42] explained that if the synthesis rate is lower than the decomposition rate of peroxides, a decrease in the peroxide index will occur, but it is faster in the production processes. However, the samples with treatments presented significantly low values, unlike the control samples.

**Table 4:** Antioxidant activity obtained by incorporating encapsulated clove essential oil in meat and meat products.

Matrix	Main Compounds of EO	Method of Analysis	Tested Concentration	Storage	Encapsulant	Encapsulation Form	Results	Ref.
Direct Addition								
Product: 57% lean beef and 10% swine fat Presentation: hamburger	Eugenol (56.06%), caryophyllene (39.63%), and α-caryophyllene (4.31%)	EO: TPC, DPPH, hydroxyl radical, and nitric oxide radicals Matrix: NR	0.304, 0.0239, and 0.0071 mg/mL (CEO particles and free CEO)	4 °C for 15 days	Sodium alginate, glycerol monostearate, and polyoxyethylene sorbitan monolaurate (Tween 20)	Solution of sodium alginate (2%) + emulsifiers glycerol monostearate (0.5%) or Tween 20 (0.5%) + CEO (1%) was homogenised in Ultra-Turrax at 12,000 rpm for 10 min. This solution was added to a calcium chloride solution (5%) while stirring in Ultra-Turrax. Particles were washed and dried at 30 °C for 48 h.	EO: -TPC = 9.07 mg GAE/g (the data were lower than reported values). -For CEO: DPPH = 94.86% for 484.7 μg/mL and 28.83% and 22.13% for hydroxyl and nitric oxide radicals, respectively, for 12.25 µg/mL. -AO particles (sodium alginate and CEO, DPPH = 9.73% and Hidroxyl = 8.31%) and AMO (sodium alginate, glycerol monostearate and CEO, DPPH = 7.69% and Hidroxyl = 7.00%) showed higher antioxidant activity for all tested radicals. Matrix: NR	[53]
Edible Films and Coatings								
Product: pork Presentation: slices of Chinese bacon (preserved meat products)	Eugenol, eugenyl acetate, and β-caryophyllene	EO: DPPH and PCL Matrix: TBAR, PV, PCL, and DPPH	0.05, 0.1, 0,2, 0.5, 1, and 2% (microcapsules)	4 °C for 3 days and then at 40 °C for 15 days	β-cyclodextrin and β-cyclodextrin metal organic frameworks (β-CD-MOFs)	1 mg/mL of the CEO solution was prepared by dissolving the oil in 45% ethanol. The oil solution was then added and dispersed into the β-CD-MOFs solution dropwise, with stirring at 48 °C to ensure thorough mixing and optimum inclusion. The mixture was cooled and freeze-dried to obtain the white powdered inclusion complex product.	EO: -All antioxidants showed similar behaviour. As the concentration of CEO increased, the free radical scavenging activity also increased. -The scavenging activity for superoxide radicals increased by 20.74%, while for hydroxyl radicals (DPPH), it was 12.84%. Matrix: -Both the MDA content and the peroxide values in each CEO group were below the permitted limit according to the national food safety standards of the People’s Republic of China: 2.5 mg/kg for animal fat and 0.5 g/100 g, respectively. -The 2% CEO concentration mostly inhibited lipid oxidation, confirming that the higher the concentration of CEO, the greater the degree of lipid inhibition. Although there was no reduction in PV in this study, it was noted that the storage temperature accelerated the lipid oxidation rate. -The samples stored at 40 °C showed thermal stability of 50.23%, indicating that microencapsulation helped protect against thermal degradation.	[67]
Product: chicken Presentation: fillets	NR	EO: NR Matrix: DPPH	2 mL Nanoparticles, 1, 3, and 5% (*w/w*)	8 ±1 °C for 6 days	Chitosan and zinc monoxide nanoparticles	0.5 g of chitosan was dissolved in 100 mL of acetic acid solution (2%). To this, 30 mL of zinc acetate solution (0.68 M) was added and stirred for 15 min. After that, 2 mL of CEO and 0.2 g of sodium tripolyphosphate were added whilst stirring. The precipitate was centrifuged (6000 rpm for 30 min), washed, and dried at 60 °C for 4 h.	EO: -The film containing the highest amount of CEO nanoparticles exhibited greater antioxidant activity, improving it by 35%, 40% and 45% compared to the control samples during the research period. Matrix: -The screening test revealed that the films with a higher content of CEO particles had greater antioxidant capacity.	[38]
Product: beef chops Presentation: pieces	Eugenol (63.4%), caryophyllene (16%), and eugenyl acetate (13.1%)	EO: DPPH Matrix: DPPH	1.7, 3.3, 6.7 and 13 μg/mL (CEO and CEO free particles)	4 °C for 12 days	Chitosan and myristic acid	Chitosan-myristic acid nanogel was prepared via the formation of amide bonds between myristic acid and chitosan through an ethylene dichloride-mediated reaction. CEO was dissolved in ethanol (1:1, *v/v*), and mixtures of the chitosan-myristic acid nanogel (10,000 mg/L) and the CEO (5000 mg/L) were prepared by sonication (70 kHz) for 5 min.	EO: -DPPH assay revealed that after 1 h, the coating containing encapsulated CEO at its highest concentration (13 µg/mL) increased by 40%, while the films with free CEO reached a value of 8.3%. -At the end of the 3 h, both free CEO and encapsulated particles (both at their maximum concentration) reached values of 65% and 100%, respectively. It is important to note that after 3 h, all groups with encapsulated particles reached 100%. Matrix: -The coatings were effective in preserving the natural colour of the meat as there was no significant change in this attribute during the research period.	[54]

NR: Not reported; DPPH: Capacity of antioxidants to scavenge DPPH (1,1-diphenyl-2-picrylhydrazil) radicals; PCL: photo-chemiluminescence; TBARS: Thiobarbituric acid reactive substances; PV: peroxide value; TPC: Total phenolic content; GAE: Gallic acid equivalent; and MDA: Malondialdehyde.

## 7. Discussion

When added directly, the CEO exhibited varying degrees of antioxidant activity and modified the organoleptic properties in meat and meat products, depending on the type of meat, concentration, and storage conditions. In beef hamburgers, CEO rich in eugenol (82.50%) was added at concentrations of 250–500 mg/kg, resulting in MDA contents below 0.5 mg/kg after three months at −18 °C. The sensory evaluation showed that samples with 250 mg/kg CEO scored above 8 in taste and odour without significant differences compared to the controls, while 500 mg/kg CEO imparted a strong clove flavour that reduced acceptability [34]. In beef mortadella treated with 0.066% CEO (80.67% eugenol), no significant differences in TBARS values were observed compared to the controls, although colour variations remained below the perceptible threshold of 3.0 [48]. In ground pork mixed with chicken thighs, a 0.00715% CEO application (the main compounds were not reported), combined with thyme oil, showed minimal antioxidant effects as the reduction in TBARS during storage at 4 and 35 °C was insignificant [45]. Red tilapia fish patties treated with 0.1% CEO maintained TBARS values below 1 mg MDA/kg, independent of the cooking method, although no sensory data were available [55]. In bonito fish patties, the addition of CEO at 2.65 L/kg led to TBARS values of 3.02 mg MDA/kg, TVB-N of 13.41 mg/100 g, and PV of 2.53 mmol O_2_/kg fat after 10 days at 4 °C. Despite maintaining acceptable appearance and texture, the samples exhibited low scores for taste and overall acceptability [40].

Chicken breast nuggets treated with 600 ppm CEO, composed mainly of eugenol (55.66%), caryophyllene (25.21%), humulene (5.32%), and δ-cadinene (5.07%), showed a 95.21% DPPH inhibition rate and an 18.53% reduction in TBARS to 1.06 mg MDA/kg after 90 days at −18 °C. The sensory evaluation indicated acceptable scores across all parameters, with overall acceptability comparable to the control [22]. Chicken burgers prepared from meat with myopathies and treated with 0.01% CEO displayed ABTS scavenging activities between 84.27% and 89.07% and TBARS values ranging from 1.88 to 2.28 mg MDA/kg, but no sensory data were reported [51]. In chicken sausages, the addition of 0.25% CEO achieved a TPC of 757.49 mg/g and TBARS values of 0.34 mg/kg after 45 days at −18 °C, although the antioxidant effect was not the most effective amongst the treatments [60]. In another chicken sausage study, despite a TBARS index of 0.9 mg MDA/kg and the highest phenolic content observed, sensory deterioration began on day 5, with losses in texture, flavour, and juiciness [59]. In turkey hamburgers, CEO containing eugenol (83.8%) and eugenol acetate (5.2%) at 1% exhibited good antioxidant activity (DPPH IC50 = 2.05 mg/mL; ABTS IC50 = 5.20 mg/mL) [47]. Buffalo meat hamburgers treated with 0.1% CEO (59.97% eugenol and 15.36% β-caryophyllene) showed a TBARS value of 0.59 mg MDA/kg after nine days at 8 °C, with oxidation levels reduced by 73% compared to the controls, but no sensory data were reported [63]. In ground sheep meat, the application of 0.25% CEO led to moderate antioxidant effects. While samples maintained good colour and general acceptability, they scored poorly in odour at the end of the nine-day storage period [35].

The incorporation of CEO into edible films and coatings demonstrated varied antioxidant effectiveness across meat and meat products. The application of CEO in beef products improved oxidative stability and extended shelf life. In beef slices coated with poly(lactic acid)/chitosan films containing CEO (eugenol, eugenol acetate and β-caryophyllene), radical scavenging activity increased by 43%, without organoleptic migration [62]. In traditional Turkish sucuk slices, coatings with 1.5% CEO (cymol 26.29% and α-pinene 20.65% and eugenol 17.02%) delayed colour deterioration and maintained TBARS at 2.00 mg MDA/kg, though water activity was negatively affected [58]. Beef fillets coated with CEO and eugenol biofilms (eugenol 67.6% and aceteugenol 16.8%) showed 87–92% radical scavenging, and lipid oxidation remained low (0.966 mg MDA/kg) after 14 days [49]. Another study reported that 3% CEO (eugenol 83.3%, caryophyllene 10.6%) reduced TBARS to 1.32 mg MDA/kg, although redness and sensory scores declined, reaching rejection levels by day 12 [52].

Edible films and coatings containing CEO also improved oxidative stability in pork matrices. In pork belly pieces, films with 0.75% CEO reduced peroxide value from 22 to 16 mEq/kg after 15 days, achieving approximately 31% DPPH and 61% ABTS radical elimination [56]. In pork patties treated with 6400 μg/mL CEO (eugenol 75%), TBARS increased up to 3.27 mg MDA/kg during storage, but the clove aroma maintained acceptable sensory scores for up to 12 days despite discolouration [65]. In pork sausages coated with CEO (eugenol 85%), TBARS remained below 1 mg MDA/kg without significant pH or water activity changes; however, flavour and odour were negatively impacted [64]. In cooked pork sausages, CEO coatings (eugenol 75%) lowered PV and TBARS values, but sensory acceptability dropped below a score of 5 after 20 days [46].

In fish matrices, CEO treatments were effective in delaying spoilage. In Pacific mackerel fillets, 2–3% CEO coatings reduced TVB-N values to 20.61–16.28 mg/100 g, although TBARS reached critical levels by day 8, and the sensory analysis was interrupted due to strong putrid odours [42]. Atlantic salmon fillets treated with 5% CEO coatings kept TBARS below 1 mg MDA/kg and extended shelf life by 5 days [68]. In Wuchang bream fillets, 1–1.5% CEO coatings lowered TBARS (1.26–1.70 mg MDA/kg) and TVB-N (23.5–27.4 mg/100 g), with acceptable sensory scores (final score of 4) despite decreasing firmness over time [50]. Tambaqui fillets coated with CEO maintained MDA values between 0.75 and 0.80 mg/kg after 120 days, with neutral sensory evaluations (scores of 4.7–5.4) [66]. In rainbow trout, coatings with 2% CEO (eugenol 77.57%) kept TBARS at 1.7 mg MDA/kg and TVB-N below 30 mg/100 g, extending shelf life to more than 12 days; however, sensory scores dropped sharply after day 8 [36].

In chicken breast fillets, CEO (eugenol 79.4%) combined with lemon EO maintained TBA at 0.54 mg MDA/kg at day 5, but higher CEO concentrations decreased sensory scores for flavour and texture [41]. Chicken burgers coated with 0.5–1% CEO films showed a TBARS of 0.98 mg MDA/kg and TVB-N of 22.8 mg/100 g after 35 days, with 0.5% CEO achieving the best sensory acceptance [61]. In ground camel meat (eugenol 76.07%), CEO films improved phenolic content by up to 45.21 mg GAE/g and maintained TBARS at 1.03 mg MDA/kg, but sensory scores were low (5.4) [21]. Buffalo meat fillets showed no significant TBARS reduction with CEO treatment but maintained good colour and acceptable odour despite an initial strong clove aroma [25]. Grass carp fillets coated with CEO (eugenol 44.9%) exhibited a 65% inhibition of cathepsin B activity, preserving texture, although no sensory evaluation was reported [69].

The results demonstrate the antioxidant potential of encapsulated CEO in various meat matrices, utilising different methods and evaluating its effectiveness under specific storage conditions. The main compounds identified in the CEO were eugenol, caryophyllene, and eugenyl acetate, although their specific concentrations varied depending on the study [53,54,67]. In beef, specifically a 57% lean beef and 10% swine fat hamburger mix, the direct addition of CEO, encapsulated in sodium alginate with glycerol monostearate and polyoxyethylene sorbitan monolaurate, showed promising antioxidant activity. The principal compounds of the EO in this application were eugenol (56.06%), caryophyllene (39.63%), and α-caryophyllene (4.31%). While the TPC of the EO was 9.07 mg GAE/g, which was lower than some reported values, the CEO exhibited a DPPH radical scavenging activity of 94.86% at 484.7 μg/mL. The encapsulated CEO also demonstrated scavenging activity against hydroxyl and nitric oxide radicals. Notably, the encapsulated particles showed higher antioxidant activity compared to free CEO, suggesting that encapsulation enhances the effectiveness of CEO [53]. The application of encapsulated CEO in pork, specifically in slices of Chinese bacon, revealed that increasing the concentration of CEO enhanced free radical scavenging activity. A 2% concentration of CEO notably inhibited lipid oxidation, keeping both MDA content and peroxide values within the national food safety limits of the People’s Republic of China. The microencapsulation process also offered protection against thermal degradation, with samples stored at 40 °C showing a thermal stability of 50.23% [67]. In chicken fillets, chitosan/zinc monoxide nanoparticles incorporating CEO within chitosan/pullulan nanocomposite films demonstrated that a higher concentration of CEO nanoparticles led to greater antioxidant activity. Specifically, the film with the highest amount of CEO nanoparticles improved antioxidant activity by 35%, 40%, and 45% compared to the control samples over the research period [38]. In beef chops, coatings containing encapsulated CEO in chitosan/myristic acid were effective in preserving the natural colour of the meat and exhibited significant antioxidant activity. The main compounds of the essential oil used in this study were eugenol (63.4%), caryophyllene (16%), and eugenyl acetate (13.1%). The DPPH assay showed that encapsulated CEO at the highest concentration (13 μg/mL) increased antioxidant activity by 40% after 1 h, compared to 8.3% for free CEO. After 3 h, the encapsulated particles reached 100% antioxidant activity [54].

The use of CEO in meat and meat products offers important benefits, such as inhibiting lipid oxidation, prolonging shelf life and improving the sensory stability of the product. Furthermore, its application reduces the use of synthetic preservatives, responding to consumer preference for natural foods. CEO also provides additional biological properties and can be incorporated using various technological methods. Among the main disadvantages is the risk of negatively altering the sensory properties of meat due to the intense aroma and flavour of clove, especially at high concentrations. The lack of standardisation in the methodologies and doses used in the studies limits the comparability of the results. Despite promising preliminary data, certain factors currently limit the industrial application of EOs as food additives. The first aspect is the intrinsic variability of EOs’ chemical composition [95]. Further research should investigate the standardisation of production and extraction processes in the field that would allow an EO with a constant and reproducible chemotype to be obtained within a given composition range. This process will also have to include an assessment of production costs. Finally, there are regulatory aspects to consider. Most essential oils, including CEO, are generally recognised as safe (GRAS) by the US Food and Drug Administration (FDA) [96], but further research is needed on nanoemulsions, coatings, and edible films that can be developed to improve the sensory properties of final meat products.

## 8. Conclusions

The studies selected in this systematic review describe an extensive scientific investigation into the use of *Syzygium aromaticum* essential oil as a plant-derived antioxidant in meat and meat products. This review stands out for its systematic approach based on the PRISMA 2020 methodology, ensuring transparency and rigour in the study selection process. The exhaustive search of multiple databases, careful filtering using clear inclusion and exclusion criteria, and the extraction of relevant variables such as meat type, clove essential oil concentration, and storage conditions strengthen the quality of the analysis. Furthermore, the review offers a comprehensive and up-to-date overview of the antioxidant potential of clove essential oil in meat and meat products.

The results show that clove essential oil, rich in eugenol, exhibits variable antioxidant effects in meat products, influenced by the type of meat, applied concentration, and storage conditions. Overall, formulations with clove essential oil were able to reduce thiobarbituric acid reactive substance levels and maintain oxidative stability, particularly in beef burgers, chicken nuggets, and buffalo meat burgers. However, the sensory impact was inconsistent: while moderate concentrations preserved sensory acceptability, higher doses led to strong and undesirable flavours, as observed in beef burgers and fish products. Additionally, some treatments, such as those applied to ground pork with chicken thighs or chicken sausages, showed limited or unstable antioxidant effects, accompanied by early sensory deterioration. These findings highlight the need to carefully adjust clove essential oil concentration and consider the specific meat matrix to maximise its antioxidant efficacy without compromising the organoleptic properties of the final product.

The use of clove essential oil in edible films and coatings demonstrated differentiated antioxidant performance depending on the meat matrix and clove essential oil composition and concentration. In beef products, clove essential oil-based films significantly enhanced oxidative stability, as evidenced by increased radical scavenging activity and reduced thiobarbituric acid reactive substance values. However, excessive concentrations led to sensory deterioration and colour loss in some cases, emphasising the need for optimised dosing strategies. In pork matrices, clove essential oil incorporation reduced lipid peroxidation and preserved physicochemical parameters. Nevertheless, sensory quality was often compromised over time, especially in sausages, indicating a trade-off between oxidative protection and organoleptic acceptability. For fish species, clove essential oil coatings delayed spoilage and maintained low thiobarbituric acid reactive substance and total volatile basic nitrogen values, particularly in Atlantic salmon, rainbow trout, and tambaqui fillets. Still, sensory scores frequently declined after extended storage, often due to the development of strong or off-putting odours, highlighting a limitation for long-term preservation. In chicken products, moderate clove essential oil concentrations proved effective in controlling lipid oxidation while maintaining acceptable sensory attributes, especially in chicken burgers. Higher clove essential oil levels, however, negatively affected flavour and texture. In camel and buffalo meat, clove essential oil films improved phenolic content and colour preservation, but the strong clove aroma and modest sensory acceptance in camel meat suggest limited consumer appeal. Altogether, clove essential oil-enriched coatings offer a promising natural strategy for extending shelf life and reducing oxidation in a wide range of meat products. Nevertheless, their practical application requires careful formulation to balance antioxidant efficacy with sensory acceptability for each specific matrix.

The incorporation of encapsulated clove essential oil into various meat matrices has demonstrated significant antioxidant potential, largely attributed to its main constituents such as eugenol, caryophyllene, and eugenyl acetate. Encapsulation strategies, using materials such as sodium alginate, chitosan, and nanocomposites, consistently enhanced the stability and efficacy of clove essential oil compared to direct addition. In beef-based products, e.g., hamburgers and beef chops, encapsulated clove essential oil significantly improved radical scavenging activity and lipid stability. Notably, encapsulation enhanced antioxidant performance over time, far surpassing free clove essential oil. In addition, colour preservation in beef chops highlights the role of encapsulation in maintaining sensory quality. In pork applications, particularly Chinese bacon, increased clove essential oil concentrations led to a greater inhibition of lipid oxidation, keeping peroxide and malondialdehyde levels within safety standards. Encapsulation also improved thermal stability, making the clove essential oil more suitable for storage under elevated temperatures. In chicken fillets, clove essential oil encapsulated in chitosan/pullulan nanocomposite films with zinc oxide nanoparticles demonstrated dose-dependent antioxidant effects. The results confirm the synergistic potential of nanotechnology and natural antioxidants. The encapsulation technologies not only preserved the bioactive compounds of clove essential oil but also significantly enhanced its antioxidant efficacy in meat systems. These findings support the development of advanced delivery systems for clove essential oil in order to improve meat product stability while maintaining food safety and quality under various storage conditions.

In light of these findings, several lines of research can be proposed to complement current studies. (I) New research should investigate the action of microencapsulated essential oil blends that also contain *S. aromaticum* essential oil. Preliminary studies on essential oil blends with a pleasant sensory profile, which may be more acceptable to consumers, could subsequently be tested to verify whether oxidation and shelf-life parameters are also improved. (II) The treatment of meat products with volatile organic compounds of *S. aromaticum* could be explored as a new line of research, considering that the treatment called ‘fumigation by essential oil’ could be less invasive in relation to organoleptic characteristics and, at the same time, produce antioxidant effects.

## Figures and Tables

**Table 1:** Keywords used for the process of finding relevant literature.

Criterion	Eligibility	Exclusion
Literature type	Journal (research articles)	Book, book series, chapter in a book, systematic review article or conference proceeding
Language	English	Non-English
Timeline	Between 1999 and 2024	1998 and earlier
Country/territory	World	

## Supplementary Materials

The following supporting information can be downloaded at: https://www.mdpi.com/article/10.3390/plants14131958/s1, Table S1: Reference list of all papers included in the review.

## Abbreviations

The following abbreviations are used in this manuscript:

β-CD	β-cyclodextrin
β-CD-MOFs	β-cyclodextrin metal organic frameworks
ε-PL	ε-polylysine
ABTS	2,2′-Azino-bis(3-ethylbenzothiazoline-6-sulfonate)
CEO	Clove essential oil
CMC	Carboxymethyl chitosan
DPPH	1,1-diphenyl-2-picrylhydrazil
DTNB	5,5′-dithiobis(2-nitribenzoic acid
EO	Essential oil
EOs	Essential oils
FAC	Fractional antioxidant capacity
FDA	Food and Drug Administration
FFA	Free fatty acids
FRAP	Ferric-reducing antioxidant power
GAE	Gallic acid equivalent
GRAS	Generally recognised as safe
MAE	Microwave-assisted extraction
MDA	Malondialdehyde
MetMb	Metmyoglobin
ORAC	Oxygen radical absorbance capacity
PCL	Polycaprolactone
PG	Propyl gallate
PS/EVOH/PE	Polystyrene/ethylene-vinyl alcohol/polyethylene
PV	Peroxide value
ROS	Reactive oxygen species
TBA	Thiobarbituric acid
TBARS	Thiobarbituric acid reactive substances
TPC	Total phenolic content
TVN	Total volatile nitrogen
TVB-N	Total volatile basic nitrogen
UAE	Ultrasound-assisted extraction
VC	Vitamin C
