# Supplementary material for: Antioxidant Application of Clove (Syzygium aromaticum) Essential Oil in Meat and Meat Products: A Systematic Review
Source: Plants (Basel). 2025 Jun 26;14(13):1958. doi: 10.3390/plants14131958 (PMC12252223; doi:10.3390/plants14131958)
Supplement: Supplementary file 1 [file plants-14-01958-s001.zip › plants-3677964-supplementary.pdf]

Table S1. Reference list of all papers included in the review.

Reference	Reference Number
Abdel-Aziz, M.E.; Morsy, N.F.S. Keeping Quality of Frozen Beef Patties by Marjoram and Clove Essential Oils. *J. Food Process. Preserv.* **2015**, *39*, 956-965. https://doi.org/10.1111/jfpp.12309.	34
Aliakbarlu, J.; Khalili Sadaghiani, S. Effect of Avishane Shirazi (Zataria Multiflora) and Clove (Syzygium Aromaticum) Essential Oils on Microbiological, Chemical and Sensory Properties of Ground Sheep Meat During Refrigerated Storage. *J. Food Qual.* **2015**, *38*, 240-247. https://doi.org/10.1111/jfq.12147.	35
Ansarian, E.; Aminzare, M.; Hassanzad Azar, H.; Mehrasbi, M.R.; Bimakr, M. Nanoemulsion-based basil seed gum edible film containing resveratrol and clove essential oil: In vitro antioxidant properties and its effect on oxidative stability and sensory characteristic of camel meat during refrigeration storage. *Meat Sci.* **2022**, *185*, 108716. https://doi.org/10.1016/j.meatsci.2021.108716.	21
Binsi, P.K.; Ninan, G.; Ravishankar, C.N. Effect of curry leaf and clove bud essential oils on textural and oxidative stability of chill stored sutchi catfish fillets. *J. Texture Stud.* **2017**, *48*, 258-266. https://doi.org/10.1111/jtxs.12237.	23
Dehghani, P.; Hosseini, S.M.H.; Golmakani, M.-T.; Majdinasab, M.; Esteghlal, S. Shelf-life extension of refrigerated rainbow trout fillets using total Farsi gum-based coatings containing clove and thyme essential oils emulsions. *Food Hydrocoll.* **2018**, *77*, 677-688. https://doi.org/10.1016/j.foodhyd.2017.11.009.	36
Echeverría, I.; López-Caballero, M.E.; Gómez-Guillén, M.C.; Mauri, A.N.; Montero, M.P. Active nanocomposite films based on soy proteins-montmorillonite- clove essential oil for the preservation of refrigerated bluefin tuna (Thunnus thynnus) fillets. *Int. J. Food Microbiol.* **2018**, *266*, 142-149. https://doi.org/10.1016/j.ijfoodmicro.2017.10.003.	37
Gasti, T.; Dixit, S.; Hiremani, V.D.; Chougale, R.B.; Masti, S.P.; Vootla, S.K.; Mudigoudra, B.S. Chitosan/pullulan based films incorporated with clove essential oil loaded chitosan-ZnO hybrid nanoparticles for active food packaging. *Carbohydr. Polym.* **2022**, *277*, 118866. https://doi.org/10.1016/j.carbpol.2021.118866.	38
Ghasemi, B.; Varidi, M.J.; Varidi, M.; Kazemi-Taskooh, Z.; Emami, S.A. The effect of plant essential oils on physicochemical properties of chicken nuggets. *J. Food Meas. Charact.* **2022**, *16*, 772-783. https://doi.org/10.1007/s11694-021-01204-1.	22
Gómez-Estaca, J.; López de Lacey, A.; López-Caballero, M.E.; Gómez-Guillén, M.C.; Montero, P. Biodegradable gelatin–chitosan films incorporated with essential oils as antimicrobial agents for fish preservation. *Food Microbiol.* **2010**, *27*, 889-896. https://doi.org/10.1016/j.fm.2010.05.012.	39
Guran, H.S.; Oksuztepe, G.; Coban, O.E.; Incili, G.K. Influence of different essential oils on refrigerated fish patties produced from bonito fish (Sarda sarda Bloch, 1793). *Czech J. Food Sci.* **2015**, *33*, 37-44. https://doi.org/10.17221/188/2014-CJFS.	40
Harmankaya, S.; Harmankaya, A.; İşbarali, K.; Paksoy, Ö.İ. Effect of rosemary and clove essential oils on lipid oxidation, microbial, sensorial properties and storage stability of kavurma, a cooked meat product. *Czech J. Food Sci.* **2024**, *42*, 251-262. https://doi.org/10.17221/56/2024-CJFS	72
Hosseini, M.; Jamshidi, A.; Raeisi, M.; Azizzadeh, M. Effect of sodium alginate coating containing clove (Syzygium Aromaticum) and lemon verbena (Aloysia Citriodora) essential oils and different packaging treatments on shelf life extension of refrigerated chicken breast. *J. Food Process. Preserv.* **2021**, *45*, e14946. https://doi.org/10.1111/jfpp.14946.	41
Hu, J.; Xu, Y.; Majura, J.J.; Qiu, Y.; Ding, J.; Hatab, S.; Miao, W.; Gao, Y. Combined Effect of the Essential Oil and Collagen Film on the Quality of Pacific Mackerel (Pneumatophorus japonicus) Fillet During Cold Storage. *Foodborne Pathog. Dis.* **2021**, *18*, 455-461. https://doi.org/10.1089/fpd.2021.0007.	42

Huang, Y.; Huang, H.; Wu, J.; Feng, Q.; Li, Y.; Li, Q.; Sun, T. Preparation and properties of PCL coaxial electrospinning films with shell loaded with CEO and core coated LEO nanoemulsions. *Food Res. Int.* **2024**, *194*, 114817. https://doi.org/10.1016/j.foodres.2024.114817.	43
Jalali, N.; Ariiai, P.; Fattahi, E. Effect of alginate/carboxyl methyl cellulose composite coating incorporated with clove essential oil on the quality of silver carp fillet and Escherichia coli O157:H7 inhibition during refrigerated storage. *J. Food Sci. Technol.* **2016**, *53*, 757-765. https://doi.org/10.1007/s13197-015-2060-4.	44
Khodaei, N.; Houde, M.; Bayen, S.; Karboune, S. Exploring the synergistic effects of essential oil and plant extract combinations to extend the shelf life and the sensory acceptance of meat products: multi-antioxidant systems. *J. Food Sci. Technol.* **2023**, *60*, 679-691. https://doi.org/10.1007/s13197-022-05653-4.	45
Lekjing, S. A chitosan-based coating with or without clove oil extends the shelf life of cooked pork sausages in refrigerated storage. *Meat Sci.* **2016**, *111*, 192-197. https://doi.org/10.1016/j.meatsci.2015.10.003.	46
Loizzo, M.R.; Tundis, R.; Menichini, F.; Duthie, G. Anti-rancidity effect of essential oils, application in the lipid stability of cooked turkey meat patties and potential implications for health. *Int. J. Food Sci. Nutr.* **2015**, *66*, 50-57. https://doi.org/10.3109/09637486.2014.953454.	47
Martins, H.H.d.A.; Simões, L.A.; Isidoro, S.R.; Nascimento, S.d.S.; Alcântara, J.P.; Ramos, E.M.; Piccoli, R.H. Preservative of Essential Oil Blends: Control of Clostridium perfringens Type a in Mortadella. *Braz. Arch. Biol. Technol.* **2021**, *64*, e21200106. https://doi.org/10.1590/1678-4324-2021200106.	48
Naveena, B.M.; Muthukumar, M.; Sen, A.R.; Babji, Y.; Murthy, T.R.K. Improvement of shelf-life of buffalo meat using lactic acid, clove oil and vitamin C during retail display. *Meat Sci.* **2006**, *74*, 409-415. https://doi.org/10.1016/j.meatsci.2006.04.020.	25
Navikaite-Snipaitiene, V.; Ivanauskas, L.; Jakstas, V.; Rüegg, N.; Rutkaite, R.; Wolfram, E.; Yildirim, S. Development of antioxidant food packaging materials containing eugenol for extending display life of fresh beef. *Meat Sci.* **2018**, *145*, 9-15. https://doi.org/10.1016/j.meatsci.2018.05.015.	49
Nisar, T.; Yang, X.; Alim, A.; Iqbal, M.; Wang, Z.-C.; Guo, Y. Physicochemical responses and microbiological changes of bream (Megalobrama ambycephala) to pectin based coatings enriched with clove essential oil during refrigeration. *Int. J. Biol. Macromol.* **2019**, *124*, 1156-1166. https://doi.org/10.1016/j.ijbiomac.2018.12.005.	50
Pinto, L.A.d.M.; Razente, R.A.; Benito, C.E.; Gubert, L.; Stefanello, L.R.; Simões, E.P.; Júnior, R.C.d.S.; Monteschio, J.d.O.; Fernandes, J.I.M. Clove essential oil (Syzygium aromaticum L.) as a natural preservative to improve the shelf-life of chicken patties with different degrees of myopathy. *J. Food Process. Preserv.* **2022**, *46*, e17037. https://doi.org/10.1111/jfpp.17037.	51
Radha krishnan, K.; Babuskin, S.; Rakhavan, K.R.; Tharavin, R.; Azhagu Saravana Babu, P.; Sivarajan, M.; Sukumar, M. Potential application of corn starch edible films with spice essential oils for the shelf life extension of red meat. *J. Appl. Microbiol.* **2015**, *119*, 1613-1623. https://doi.org/10.1111/jam.12932.	52
Radünz, M.; da Trindade, M.L.M.; Camargo, T.M.; Radünz, A.L.; Borges, C.D.; Gandra, E.A.; Helbig, E. Antimicrobial and antioxidant activity of unencapsulated and encapsulated clove (Syzygium aromaticum, L.) essential oil. *Food Chem.* **2019**, *276*, 180-186. https://doi.org/10.1016/j.foodchem.2018.09.173.	53
Rajaei, A.; Hadian, M.; Mohsenifar, A.; Rahmani-Cherati, T.; Tabatabaei, M. A coating based on clove essential oils encapsulated by chitosan-myristic acid nanogel efficiently enhanced the shelf-life of beef cutlets. *Food Packag. Shelf Life* **2017**, *14*, 137-145. https://doi.org/10.1016/j.fpsl.2017.10.005.	54
Ramezani-Fard, E.; Romano, N.; Goh, Y.-M.; Oskoueian, E.; Ehteshami, F.; Ebrahimi, M. The effect of different cooking methods on fatty acid composition and antioxidant activity of n-3	55

fatty acids fortified tilapia meat with or without clove essential oil. *J. Environ. Biol.* **2016**, *37*, 775-784.	
Roy, S.; Priyadarshi, R.; Rhim, J.-W. Gelatin/agar-based multifunctional film integrated with copper-doped zinc oxide nanoparticles and clove essential oil Pickering emulsion for enhancing the shelf life of pork meat. *Food Res. Int.* **2022**, *160*, 111690. https://doi.org/10.1016/j.foodres.2022.111690.	56
Salgado, P.R.; López-Caballero, M.E.; Gómez-Guillén, M.C.; Mauri, A.N.; Montero, M.P. Sunflower protein films incorporated with clove essential oil have potential application for the preservation of fish patties. *Food Hydrocoll.* **2013**, *33*, 74-84. https://doi.org/10.1016/j.foodhyd.2013.02.008.	57
Saricaoglu, F.T.; Turhan, S. Performance of mechanically deboned chicken meat protein coatings containing thyme or clove essential oil for storage quality improvement of beef sucuks. *Meat Sci.* **2019**, *158*, 107912. https://doi.org/10.1016/j.meatsci.2019.107912.	58
Sharma, H.; Mendiratta, S.K.; Agarwal, R.K.; Kumar, S.; Soni, A. Evaluation of anti-oxidant and anti-microbial activity of various essential oils in fresh chicken sausages. *J. Food Sci. Technol.* **2017**, *54*, 279-292. https://doi.org/10.1007/s13197-016-2461-z.	59
Sharma, H.; Mendiratta, S.K.; Agrawal, R.K.; Gurunathan, K.; Kumar, S.; Singh, T.P. Use of various essential oils as bio preservatives and their effect on the quality of vacuum packaged fresh chicken sausages under frozen conditions. *LWT - Food Sci. Technol.* **2017**, *81*, 118-127. https://doi.org/10.1016/j.lwt.2017.03.048.	60
Shukla, V.; Mendiratta, S.K.; Zende, R.J.; Agrawal, R.K.; Kumar Jaiswal, R. Effects of chitosan coating enriched with Syzygium aromaticum essential oil on quality and shelf-life of chicken patties. *J. Food Process. Preserv.* **2020**, *44*, e14870. https://doi.org/10.1111/jfpp.14870.	61
Stoleru, E.; Vasile, C.; Irimia, A.; Brebu, M. Towards a Bioactive Food Packaging: Poly(Lactic Acid) Surface Functionalized by Chitosan Coating Embedding Clove and Argan Oils. *Molecules* **2021**, *26*, 4500. https://doi.org/10.3390/molecules26154500.	62
Tajik, H.; Farhangfar, A.; Moradi, M.; Razavi Rohani, S.M. Effectiveness of Clove Essential Oil and Grape Seed Extract Combination on Microbial and Lipid Oxidation Characteristics of Raw Buffalo Patty During Storage at Abuse Refrigeration Temperature. *J. Food Process. Preserv.* **2014**, *38*, 31-38. https://doi.org/10.1111/j.1745-4549.2012.00736.x.	63
Ugalde, M.L.; de Cezaro, A.M.; Vedovatto, F.; Paroul, N.; Steffens, J.; Valduga, E.; Backes, G.T.; Franceschi, E.; Cansian, R.L. Active starch biopolymeric packaging film for sausages embedded with essential oil of Syzygium aromaticum. *J. Food Sci. Technol.* **2017**, *54*, 2171-2175. https://doi.org/10.1007/s13197-017-2624-6.	64
Venkatachalam, K.; Lekjing, S. A chitosan-based edible film with clove essential oil and nisin for improving the quality and shelf life of pork patties in cold storage. *RSC Advances* **2020**, *10*, 17777-17786. https://doi.org/10.1039/D0RA02986F.	65
Vieira, B.B.; Mafra, J.F.; Bispo, A.S.d.R.; Ferreira, M.A.; Silva, F.d.L.; Rodrigues, A.V.N.; Evangelista-Barreto, N.S. Combination of chitosan coating and clove essential oil reduces lipid oxidation and microbial growth in frozen stored tambaqui (Colossoma macropomum) fillets. *LWT* **2019**, *116*, 108546. https://doi.org/10.1016/j.lwt.2019.108546.	66
Wang, Y.; Du, Y.T.; Xue, W.Y.; Wang, L.; Li, R.; Jiang, Z.T.; Tang, S.H.; Tan, J. Enhanced preservation effects of clove (Syzygium aromaticum) essential oil on the processing of Chinese bacon (preserved meat products) by beta cyclodextrin metal organic frameworks (β-CD-MOFs). *Meat Sci.* **2023**, *195*, 108998. https://doi.org/10.1016/j.meatsci.2022.108998.	67
Wei, Z.; Zhang, J.; Zhang, H.; Zhang, N.; Zhang, R.; Li, L.; Liu, G. Effect of nanoemulsion loading a mixture of clove essential oil and carboxymethyl chitosan-coated ε-polylysine on the preservation of donkey meat during refrigerated storage. *J. Food Process. Preserv.* **2021**, *45*, e15733. https://doi.org/10.1111/JFPP.15733.	71

Xiong, Y.; Kamboj, M.; Ajlouni, S.; Fang, Z. Incorporation of salmon bone gelatine with chitosan, gallic acid and clove oil as edible coating for the cold storage of fresh salmon fillet. *Food Control* **2021**, *125*, 107994. https://doi.org/10.1016/j.foodcont.2021.107994.	68
Yu, D.; Regenstein, J.M.; Zang, J.; Xia, W.; Xu, Y.; Jiang, Q.; Yang, F. Inhibitory effects of chitosan-based coatings on endogenous enzyme activities, proteolytic degradation and texture softening of grass carp (Ctenopharyngodon idellus) fillets stored at 4 °C. *Food Chem.* **2018**, *262*, 1-6. https://doi.org/10.1016/j.foodchem.2018.04.070	69
Zengin, H.; Baysal, A.H. Antioxidant and Antimicrobial Activities of Thyme and Clove Essential Oils and Application in Minced Beef. *J. Food Process. Preserv.* **2015**, *39*, 1261-1271. https://doi.org/10.1111/jfpp.12344.	70
